# Peritumoral Radiomic Deviation on DCE-MRI Exceeds the Bilateral Normal-Appearing Parenchymal Distance in Breast Cancer: A Within-Patient Multi-Metric Study

**DOI:** 10.3390/cancers18203347

**Published:** 2026-10-16

**Authors:** Zeynep Hilal Kilimci, Demet Doğan, Kağan Gökçe, Özgür Çakır, Oğuzhan Urhan

**Affiliations:** 1 Department of Information Systems Engineering, Faculty of Technology, Kocaeli University, Kocaeli 41001, Türkiye; 2 Department of Radiology, Faculty of Medicine, Istanbul Okan University, İstanbul 34959, Türkiye; demet.dogan@okan.edu.tr; 3 Department of General Surgery, Faculty of Medicine, Istanbul Okan University, İstanbul 34959, Türkiye; kagan.gokce@okan.edu.tr; 4 Department of Radiology, Faculty of Medicine, Kocaeli University, Kocaeli 41001, Türkiye; ozgur.cakir@kocaeli.edu.tr; 5 Department of Electronics and Telecommunication, Faculty of Engineering, Kocaeli University, Kocaeli 41001, Türkiye; urhano@kocaeli.edu.tr; 6 KOU Teknopark, EVSTEK Bilişim Teknolojileri A.Ş., Kocaeli 41275, Türkiye

**Keywords:** breast cancer, radiomics, peritumoral microenvironment, tumor microenvironment, normal-appearing breast parenchyma, radiomic distance, within-patient reference, dynamic contrast-enhanced MRI, magnetic resonance imaging, single-slice radiomics

## Abstract

Background: The imaging-defined peritumoral region may contain quantitative imaging information beyond that captured by the tumor core, but its radiomic deviation from normal-appearing breast parenchyma remains insufficiently characterized. This retrospective single-center study investigated whether peritumoral-to-normal radiomic distances exceed the within-patient bilateral normal-appearing parenchymal reference distance. Fifty-six patients with histopathologically confirmed breast cancer and preoperative bilateral dynamic contrast-enhanced MRI were included. Methods: Single-slice radiomic analysis was performed for four predefined regions: tumor core (T), peritumoral microenvironment (M), remote ipsilateral normal-appearing parenchyma (I), and contralateral normal-appearing parenchyma (C). After quality control, 101 radiomic features were standardized and analyzed using standardized Euclidean, Manhattan, cosine, and Ledoit–Wolf Mahalanobis distances. Peritumoral-to-ipsilateral ($d_{MI}$) and peritumoral-to-contralateral ($d_{MC}$) distances were compared with the within-patient bilateral normal-appearing parenchymal distance ($d_{IC}$). Results: Across all four metrics, both $d_{MI}$ and $d_{MC}$ exceeded $d_{IC}$, and all eight primary paired Wilcoxon comparisons remained significant after Benjamini–Hochberg correction ($q\leq 1.07\times 10^{-6}$), with matched-pairs rank-biserial correlations ranging from 0.749 to 0.989. The principal pattern was broadly preserved across alternative Mahalanobis covariance formulations, reduced-dimensional PCA analyses, particularly when three or four principal components were retained, exclusion of all 13 retained shape features, separate radiomic feature-family analyses, and replacement of selected non-normalized non-uniformity features with normalized counterparts. Conclusions: These findings show that, in the present cohort, peritumoral-to-normal radiomic distances exceeded the within-patient bilateral normal-appearing parenchymal reference distance. Because the four analyzed regions differed in size, shape, and sampling geometry, the reported distances should be interpreted as region-level radiomic differences under the present ROI definitions rather than as geometry-independent tissue properties. Larger multi-institutional studies using full-volume three-dimensional radiomics and standardized imaging and preprocessing strategies are needed to assess reproducibility, clarify the biological correlates of this radiomic pattern, and evaluate its potential clinical relevance.

## Simple Summary

Tissue surrounding a breast tumor may contain quantitative imaging characteristics that differ from more remote breast parenchyma, even when these differences are not apparent by visual assessment. Quantitative image analysis, known as radiomics, can measure such alterations, but normal breast tissue is itself variable, which makes it difficult to judge how large a peritumoral change really is. In this retrospective single-center study of 56 patients with breast cancer, we measured radiomic differences between the tissue immediately surrounding the tumor and normal-appearing breast tissue in the same patient, and compared these differences with the radiomic difference between the normal-appearing tissue of the two breasts. Across four mathematically different distance measures, the peritumoral tissue differed from normal-appearing tissue more than the two normal-appearing regions differed from each other. This within-patient reference approach may offer a way to place peritumoral radiomic measurements in the context of each patient’s own tissue variability. The findings are based on single-slice analysis in a small single-center cohort and require confirmation in larger studies before any clinical use can be considered.

## 1. Introduction

Breast cancer is the most frequently diagnosed malignancy among women worldwide and remains a leading cause of cancer-related mortality. According to the Global Cancer Observatory estimates for 2022, female breast cancer accounted for approximately 2.3 million new cases and 11.6% of all newly diagnosed cancers globally [1]. Breast cancer is characterized by substantial biological, molecular, and spatial heterogeneity, and its behavior is determined not only by malignant epithelial cells but also by interactions with the surrounding tumor microenvironment (TME). The TME comprises stromal and immune cells, fibroblasts, endothelial cells, extracellular matrix components, and soluble signaling factors that collectively influence tumor growth, invasion, treatment response, and metastatic progression [2,3]. Accordingly, characterization of tissue beyond the visible tumor boundary may provide complementary information regarding tumor-associated biological processes that may not be fully captured by analysis of the tumor core alone.

Radiomics enables the quantitative characterization of medical images through the extraction of high-dimensional features describing intensity distributions, texture, shape, and spatial heterogeneity [4,5]. Recent applications across CT and MRI further illustrate the use of radiomic descriptors for non-invasive disease differentiation, biomarker-associated characterization, and feature-selection-based classification across different anatomical sites [6,7,8]. In breast cancer, magnetic resonance imaging (MRI), particularly dynamic contrast-enhanced MRI (DCE-MRI), provides a suitable platform for radiomic analysis because of its high soft-tissue contrast and its ability to depict both the primary lesion and the surrounding breast parenchyma. Although radiomic investigations initially concentrated predominantly on intratumoral regions, increasing evidence suggests that the peritumoral region may also contain clinically and biologically relevant imaging information. This concept is consistent with the biological complexity of the TME, in which stromal remodeling, vascular alterations, inflammatory processes, and extracellular matrix changes may occur in tissue adjacent to the tumor [2,3]. Radiomic characterization of the peritumoral region therefore offers an opportunity to quantitatively investigate imaging alterations beyond the tumor boundary.

Several studies have demonstrated the potential value of peritumoral radiomics in breast cancer. Braman et al. reported that combined intratumoral and peritumoral radiomic features derived from pretreatment DCE-MRI were associated with pathological complete response to neoadjuvant chemotherapy [9]. The same group subsequently demonstrated associations between peritumoral radiomic characteristics, tumor biology, pathological response to HER2-targeted therapy, and the spatial organization of tumor-infiltrating lymphocytes in HER2-positive breast cancer [10]. Li et al. showed that radiomic features extracted from intratumoral and peritumoral regions of DCE-MRI functional parametric maps contributed to the prediction of HER2 and Ki-67 status [11]. More recently, Wu et al. investigated combined intratumoral and peritumoral MRI radiomics for the assessment of programmed death-ligand 1 expression in breast cancer [12]. Collectively, these studies support the premise that the peritumoral region contains quantitative imaging information associated with molecular characteristics, immune-related features, and treatment response.

Despite this growing body of evidence, most previous studies have incorporated peritumoral radiomic features into predictive models targeting predefined molecular, pathological, or clinical endpoints. Comparatively less attention has been given to a more fundamental characterization question: how far does the radiomic phenotype of the peritumoral microenvironment deviate from normal-appearing breast tissue, and is this deviation greater than the bilateral variation already present within the same patient? This distinction is important because normal-appearing breast parenchyma is itself heterogeneous. Background parenchymal enhancement (BPE), for example, varies both between individuals and within the same individual and can be influenced by hormonal and physiological factors [13,14]. Radiomic analysis of background parenchymal enhancement has also shown that quantitative parenchymal heterogeneity may provide information complementary to tumor-derived features in breast cancer characterization [15]. Quantitative MRI studies have also used the contralateral normal-appearing breast to characterize patient-specific parenchymal properties [16]. Consequently, interpreting a peritumoral-to-normal-appearing tissue distance in isolation may not be sufficient to distinguish tumor-associated alteration from naturally occurring bilateral variation. A within-patient comparison between normal-appearing breast tissues may therefore provide an internal reference against which the magnitude of peritumoral radiomic deviation can be contextualized.

In this study, we developed an intra-patient, multi-metric radiomic distance framework to characterize the peritumoral microenvironment in breast cancer. Radiomic distances between the peritumoral microenvironment and remote ipsilateral normal-appearing breast tissue ($d_{MI}$) and between the peritumoral microenvironment and contralateral normal-appearing breast tissue ($d_{MC}$) were interpreted relative to the radiomic distance between ipsilateral and contralateral normal-appearing tissues ($d_{IC}$). The $d_{IC}$ measure was used as an internal patient-specific reference representing the radiomic distance between the selected ipsilateral and contralateral normal-appearing parenchymal regions. To determine whether the observed relationships were robust to different mathematical definitions of dissimilarity, four complementary measures—standardized Euclidean, Manhattan, cosine, and Mahalanobis distances—were applied. The distance between the tumor core and the peritumoral microenvironment ($d_{MT}$) was additionally evaluated to contextualize the position of the peritumoral region relative to the primary tumor. We hypothesized that both $d_{MI}$ and $d_{MC}$ would exceed $d_{IC}$. We note at the outset that the present analysis is deliberately descriptive and methodological in scope. Radiomic features were derived from single axial slice regions of interest, the four analyzed regions differ in size and geometry by design, and no clinical, molecular, or histopathological endpoint was evaluated. The study therefore addresses whether a measurable within-patient radiomic separation exists under a defined analytical framework, and not whether this separation reflects a specific biological process or carries clinical value.

The main contributions of this study can be summarized as follows:

- We develop an intra-patient, single-slice radiomic distance framework for quantitatively characterizing the peritumoral region relative to the patient-specific bilateral normal-appearing parenchymal reference distance.
- We use the radiomic distance between ipsilateral and contralateral normal-appearing breast tissues ($d_{IC}$) as an internal reference, enabling peritumoral deviation to be interpreted relative to the bilateral normal-appearing parenchymal reference distance within the same patient rather than as an isolated distance measure.
- We assess the robustness of peritumoral radiomic deviation using four complementary distance measures—standardized Euclidean, Manhattan, cosine, and Mahalanobis distances—thereby examining the observed relationships from geometric, absolute-difference, directional, and covariance-aware multivariate perspectives.
- We jointly characterize the tumor core, peritumoral microenvironment, ipsilateral normal-appearing tissue, and contralateral normal-appearing tissue, allowing the peritumoral region to be evaluated both in relation to normal-appearing breast tissue variation and in the context of the primary tumor radiomic phenotype.
- We evaluate peritumoral deviation using patient-level paired comparisons against the bilateral normal-appearing parenchymal reference and quantify the robustness of the observed effects using multiple-comparison-corrected statistical testing and effect-size estimates.

### Related Work

One of the early studies establishing the relevance of peritumoral radiomics in breast MRI was conducted by Braman et al. [9]. The authors retrospectively analyzed pretreatment DCE-MRI from 117 patients undergoing neoadjuvant chemotherapy and extracted 99 textural features from both intratumoral and peritumoral regions. A combined intratumoral–peritumoral model achieved an AUC of 0.74 in the independent test cohort for predicting pathological complete response (pCR), while receptor-specific analyses yielded higher performance in selected molecular subgroups. Importantly, the study demonstrated that peritumoral heterogeneity carried response-related information that was not fully represented by the tumor core, providing early evidence that quantitative imaging beyond the visible tumor boundary may capture biologically relevant tissue alterations.

Braman et al. subsequently extended this concept by investigating the relationship between peritumoral radiomic patterns, HER2-positive tumor biology, response to targeted therapy, and tumor-infiltrating lymphocytes [10]. In a study involving 209 patients across multiple cohorts, peritumoral features located approximately 9–12 mm from the tumor showed stronger discrimination of the HER2-enriched molecular subtype than intratumoral features alone, with a maximum AUC of 0.85 compared with 0.76 for intratumoral features. A model combining intra- and peritumoral information achieved an AUC of 0.89 for identifying the HER2-enriched subtype and was associated with pathological response in independent validation cohorts. Moreover, features derived from the immediate 0–3 mm peritumoral region were associated with tumor-infiltrating lymphocyte density. These findings provided direct evidence of an association between MRI-derived peritumoral radiomic features and an immune-related histopathological characteristic, namely tumor-infiltrating lymphocyte density.

Li et al. [11] investigated intratumoral and peritumoral radiomics derived from functional parametric maps of DCE-MRI for predicting HER2 and Ki-67 status in 351 patients with breast cancer. Radiomic signatures were independently constructed from intratumoral, peritumoral, and combined regions. In the validation cohort, the combined signatures achieved AUCs of 0.713 and 0.749 for HER2 and Ki-67 prediction, respectively. Although the combined models did not significantly outperform the individual-region models, the study showed that peritumoral radiomic features contained information relevant to HER2 and Ki-67 status. These results further support the use of the peritumoral region as a quantitatively informative imaging compartment rather than merely a geometric extension surrounding the lesion.

Wu et al. [12] evaluated whether intratumoral and peritumoral DCE-MRI radiomics could noninvasively characterize programmed death-ligand 1 (PD-L1) expression in 196 patients with primary breast cancer. Separate intratumoral, peritumoral, and combined radiomic signatures were developed after feature selection. In the test cohort, the peritumoral signature achieved an AUC of 0.846, while the combined intra- and peritumoral signature achieved an AUC of 0.853. Although the performance differences among the models were not statistically significant, the performance of the peritumoral signature indicated that radiomic characteristics surrounding the tumor may contain information associated with PD-L1 status.

The spatial extent used to define peritumoral tissue has also emerged as an important methodological consideration. Zhou et al. [17] analyzed multiparametric MRI from 992 patients and systematically compared intratumoral regions with peritumoral expansions of 2, 4, 6, and 8 mm for prediction of HER2 status. Radiomic features were extracted from DCE-T1, T2-weighted, and diffusion-weighted MRI. Across the evaluated sequences, 4-mm peritumoral regions frequently provided the strongest single-region performance, while the optimal multisequence peritumoral signature achieved an AUC of 0.795 in the test cohort. These results indicate that radiomic information within the peritumoral environment is spatially dependent and that the definition of the peritumoral region can materially affect the captured imaging phenotype.

In parallel with research focused directly on the tumor surroundings, evidence has accumulated that normal-appearing breast parenchyma can provide quantitative imaging information relevant to breast cancer characterization. Arefan et al. [18] quantitatively evaluated background parenchymal enhancement (BPE) in both ipsilateral and contralateral breasts using DCE-MRI and investigated its association with breast cancer recurrence risk as represented by the Oncotype DX recurrence score. Quantitative BPE measurements from both breasts were associated with a high-risk recurrence score, and combining BPE measurements with tumor radiomic features achieved AUCs of 0.94 in the development cohort and 0.79 in the internal test cohort. These findings are particularly relevant to intra-patient analyses because they demonstrate that quantitative characteristics of non-tumoral parenchyma in both breasts may contain information associated with patient-specific recurrence-risk stratification and should not automatically be treated as a homogeneous imaging reference.

Lo Gullo et al. [19] further demonstrated that radiomic information from the contralateral unaffected breast may be informative about tumor subtype in the affected breast. Their study included 541 patients and developed a machine-learning model using radiomic features extracted exclusively from fibroglandular tissue of the contralateral unaffected breast to predict triple-negative breast cancer. A four-feature model achieved an AUC of 0.71 in the validation cohort. These results indicate that quantitative imaging characteristics of contralateral fibroglandular tissue may contain information associated with tumor subtype, further supporting the importance of accounting for patient-specific parenchymal characteristics when defining an internal radiomic reference.

More recent multicenter investigations have continued to demonstrate the incremental value of peritumoral information. Cao et al. [20] analyzed 266 patients using DCE-MRI, diffusion-weighted imaging, and fat-suppressed T2-weighted imaging to predict HER2 expression status. Tumor volumes were expanded by 3 mm to obtain peritumoral regions, and multiple random-forest models were constructed from intratumoral, peritumoral, and multiparametric feature combinations. The model integrating intratumoral and peritumoral information across all three MRI sequences achieved AUCs of 0.822, 0.823, and 0.813 in the training, internal validation, and external validation cohorts, respectively. The consistent performance across the training, internal validation, and external test cohorts provides additional evidence that combined intratumoral and peritumoral information can retain predictive value across independent cohorts.

In 2026, Chen et al. [21] investigated MRI-based peritumoral radiomics for estimating the 21-gene recurrence score in ER-positive/HER2-negative breast cancer. The study included patients from two centers and evaluated tumor and peritumoral regions with radial extensions of 1, 3, 5, 7, and 9 mm. Model performance initially increased and subsequently decreased as the peritumoral thickness expanded, with the 3-mm T2-weighted peritumoral model demonstrating the best single peritumoral performance. A fusion model integrating peritumoral, intratumoral, imaging, and clinicopathological information achieved AUCs of 0.82 and 0.75 in the internal and external validation cohorts, respectively. Besides demonstrating the potential value of peritumoral radiomics for recurrence-risk stratification, this study again illustrates that peritumoral information is spatially structured and cannot necessarily be represented by an arbitrarily defined surrounding region.

Ding et al. [22] recently introduced a habitat-aware, multiscale DCE-MRI radiomics framework for preoperative prediction of axillary lymph node metastasis. Rather than representing the tumor and its surroundings as homogeneous regions, the authors delineated intratumoral habitats using superpixel-based segmentation and clustering and combined these features with concentric peritumoral signatures and clinical factors. The fusion model incorporating the habitat signature, a 1-mm peritumoral signature, and clinical variables achieved AUCs of 0.940, 0.905, 0.857, and 0.884 across the training, validation, and two independent test cohorts, respectively. These findings demonstrate the potential value of jointly incorporating intratumoral heterogeneity, peritumoral radiomic information, and clinical variables within a multiscale predictive framework.

Similarly, Luo et al. [23] developed a multicenter MRI habitat-radiomics framework for differentiating HER2-positive, HER2-low, and HER2-zero breast cancers in 515 patients from five hospitals. Their approach integrated intratumoral heterogeneity and peritumoral radiomic features, together with clinical information in the combined model. For differentiation between HER2-low and HER2-zero disease, the peritumor-plus-intratumoral-heterogeneity model achieved AUCs of 0.839 and 0.831 in the training and internal testing cohorts and 0.799 and 0.734 in the two external validation cohorts, respectively. The combined clinical–radiomics model further improved performance in the external cohorts. These findings support the complementary predictive value of integrating peritumoral information with measures of intratumoral heterogeneity.

Taken together, the existing literature shows that peritumoral radiomic features can contribute to models addressing treatment response, receptor status, immune-related biomarkers, recurrence risk, and nodal metastasis. Studies evaluating ipsilateral and contralateral parenchyma further indicate that normal-appearing breast tissue itself contains measurable patient-specific imaging information [18,19]. However, previous work has predominantly focused on predictive modeling for predefined clinical or molecular endpoints. Comparatively less attention has been directed toward characterizing the peritumoral phenotype relative to the magnitude of bilateral normal-appearing parenchymal reference distance within the same patient. The present study examines this question by using the bilateral normal-appearing parenchymal distance ($d_{IC}$) as an intra-patient internal reference and evaluating whether peritumoral deviations ($d_{MI}$ and $d_{MC}$) exceed this reference across complementary mathematical definitions of radiomic dissimilarity.

## 2. Materials and Methods

This study was designed as an intra-patient radiomic characterization analysis of breast tumor tissue, the peritumoral microenvironment, and normal-appearing breast parenchyma. For each patient, radiomic features were extracted from four predefined regions: the tumor core, the peritumoral microenvironment, remote ipsilateral normal-appearing breast tissue, and contralateral normal-appearing breast tissue. After feature preprocessing and standardization, patient-specific radiomic distances were calculated using standardized Euclidean, Manhattan, cosine, and Mahalanobis metrics. The distance between ipsilateral and contralateral normal-appearing breast tissues ($d_{IC}$) was used as the within-patient bilateral parenchymal reference, and the peritumoral-to-normal distances were evaluated relative to this reference. Statistical analyses were subsequently performed using paired patient-level comparisons, effect-size estimation, multiple-testing correction, and complementary sensitivity analyses to assess the robustness of the observed radiomic deviations.

### 2.1. Study Population and MRI Data

This retrospective, single-center study included 56 adult patients with histopathologically confirmed breast cancer who underwent preoperative dynamic contrast-enhanced breast magnetic resonance imaging (DCE-MRI) at Istanbul Okan University Hospital between January 2021 and March 2026. All patients had complete bilateral breast MRI examinations, and the four predefined radiomic regions required for the present analysis were available.

The inclusion criteria were age ≥18 years, histopathologically confirmed primary breast cancer, availability of preoperative DCE-MRI performed at the study institution, a primary lesion clearly visible on MRI, complete bilateral breast imaging, image quality suitable for radiomic analysis, availability of the required MRI sequences, and availability of the demographic and clinical information required for eligibility assessment. Patients were excluded if they had undergone definitive surgery or neoadjuvant treatment for the index breast cancer before MRI, had a history of breast radiotherapy, had poor image quality or an incomplete MRI examination, had multifocal or multicentric disease, had bilateral breast cancer, or lacked suitable normal-appearing parenchymal regions for reference ROI placement. During the predefined study period, all 112 patients with pathologically confirmed primary breast cancer who underwent preoperative DCE-MRI at the study institution were assessed for eligibility. Of these, 56 patients were excluded: 20 because they had received neoadjuvant treatment before MRI, 12 because of multifocal disease, 14 because of an incomplete MRI protocol, and 10 because of motion artifacts. The remaining 56 patients met the prespecified eligibility criteria and were consecutively included in the final study cohort.

The mean patient age was 53.75 ± 13.95 years, with a median age of 52.5 years and a range of 30–85 years. Because not all patients underwent surgery at the study institution, postoperative histopathological data were available for 47 of the 56 patients. Hormone-receptor results were available for 46 patients, and Ki-67 measurements were available for 45 patients. Detailed clinicopathological characteristics of the available data are presented in Section 3.1.

All breast MRI examinations were performed using the same 1.5-T MRI system (SIGNA Explorer; GE Healthcare, Milwaukee, WI, USA) with a dedicated bilateral breast coil and an institutional clinical breast MRI protocol. The protocol included axial T1-weighted imaging, axial fat-suppressed T2-weighted imaging, diffusion-weighted imaging (DWI), and dynamic contrast-enhanced T1-weighted imaging.

The nominal DCE-MRI acquisition parameters included a repetition time (TR) of 4.5 ms, an echo time (TE) of 1.6 ms, a slice thickness of approximately 1.0 mm, an in-plane spatial resolution of approximately 0.7×0.7 mm^2^, and an acquisition matrix of 384×384. Gadobutrol (Gadovist; Bayer HealthCare, Berlin, Germany) was administered intravenously at a dose of 0.1 mmol/kg and an injection rate of 2.0 mL/s, followed by a 20-mL saline flush. One pre-contrast phase and five post-contrast phases were acquired, with a temporal resolution of 60 s per phase.

Radiomic feature extraction was performed using the native image geometry without spatial resampling. Review of the image and mask metadata used in the finalized radiomic dataset showed an in-plane pixel spacing ranging from 0.566 to 0.879 mm and a slice thickness ranging from 0.8 to 1.8 mm across examinations. These native geometric variations were retained during feature extraction and are addressed as a methodological limitation in Section 4.6.

Radiomic feature extraction was performed from the original, non-subtracted images of the third post-contrast phase of the DCE T1-weighted series. The third post-contrast phase was selected a priori on the basis of radiological review because it provided clear visualization of tumor enhancement and lesion margins. Using the same temporal post-contrast phase for ROI delineation and radiomic feature extraction across all patients standardized the imaging time point and reduced potential phase-related variability. This temporal-phase selection is also consistent with previous multiphase breast DCE-MRI radiomics findings in which models based on third post-contrast subtraction images showed the highest overall performance [24]. All MRI examinations were reviewed before radiomic analysis to ensure sufficient image quality and adequate visualization of the four predefined tissue regions.

The study was conducted in accordance with the Declaration of Helsinki and was approved by the Istanbul Okan University Ethics Committee (meeting date: 1 April 2026; meeting no. 200; decision no. ETK26040014). The requirement for written informed consent was waived because of the retrospective nature of the study and the use of anonymized pre-existing clinical and imaging data.

### 2.2. Region-of-Interest Definition

**Figure 1:**
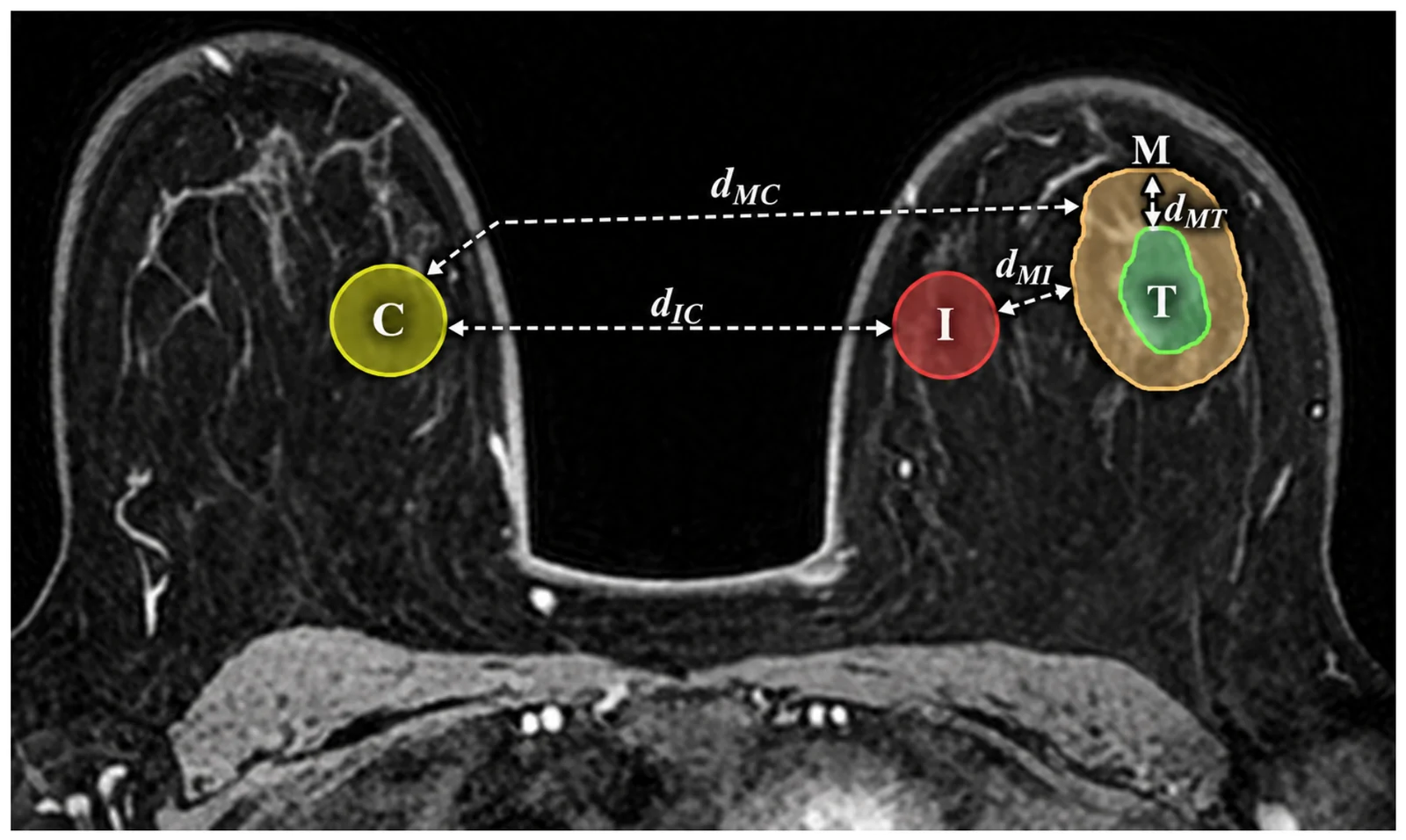
Representative annotated DCE-MRI image illustrating the four predefined regions of interest and the radiomic distance definitions used in the study. The tumor core (T) is shown in green, the peritumoral microenvironment (M) in brown, the ipsilateral normal-appearing parenchymal region (I) in red, and the contralateral normal-appearing parenchymal region (C) in yellow. The radiomic distances are defined as $d_{MT}$ between M and T, $d_{MI}$ between M and I, $d_{MC}$ between M and C, and $d_{IC}$ between I and C. The arrows are schematic representations of pairwise radiomic comparisons in feature space and should not be interpreted as physical Euclidean distances on the image.

Four anatomically distinct regions were defined for each patient to characterize the radiomic relationships among the primary tumor, its surrounding peritumoral microenvironment, and normal-appearing breast parenchyma. These regions comprised: (i) the tumor core (T), (ii) the peritumoral microenvironment (M), (iii) remote ipsilateral normal-appearing breast parenchyma (I), and (iv) contralateral normal-appearing breast parenchyma (C). A representative annotated DCE-MRI image illustrating these four ROI types and the principal radiomic distance definitions is shown in Figure 1.

The tumor region (T) corresponded to the primary breast lesion identified on MRI. Initial tumor delineation was performed volumetrically on subtraction images derived from the third post-contrast phase of the DCE-MRI series. For radiomic feature extraction, however, the ROI mask supplied to the analysis comprised a single axial slice representing the segmented tumor.

The peritumoral microenvironment (M) was generated by extending the segmented tumor boundary outward by 5 mm and excluding voxels belonging to the primary tumor from the expanded region. The 5-mm expansion was defined a priori by the supervising breast radiologist before radiomic analysis to provide a consistent sampling width for the tissue immediately surrounding the tumor across all patients. This choice was also supported by previous breast DCE-MRI radiomics work in which the peritumoral region was defined using a 2.5–5-mm radius depending on image pixel size [9]. The 5-mm width was not selected through optimization within the present cohort, and alternative peritumoral widths were not compared. Because the mask supplied for feature extraction occupied a single axial slice, the analyzed peritumoral region corresponds to an in-plane annular band surrounding the tumor cross-section rather than a three-dimensional peritumoral shell. Portions extending into the skin, pectoral muscle, chest wall, adipose tissue, or beyond the breast boundaries were excluded during ROI review. The peritumoral mask used for radiomic feature extraction was represented on a single axial slice, consistent with the single-slice radiomic analysis applied to the other predefined regions.

The ipsilateral normal-appearing parenchymal region (I) was selected from the breast ipsilateral to the primary tumor but spatially separated from the tumor and peritumoral region. A circular ROI with a diameter of 10 mm was placed within normal-appearing fibroglandular tissue without radiologically suspicious findings. Areas containing cysts, suspicious focal or asymmetric enhancement, or prominent imaging artifacts were avoided. The minimum edge-to-edge distance between the ipsilateral reference ROI and the tumor or peritumoral boundary was 20 mm.

The contralateral normal-appearing parenchymal region (C) was selected from the opposite breast within radiologically normal-appearing fibroglandular tissue. A circular ROI with a diameter of 10 mm was placed with attention to maintaining a comparable ROI size and approximately corresponding anatomical location and anterior-to-posterior depth relative to the ipsilateral reference region. Areas containing cysts, suspicious focal or asymmetric enhancement, or prominent imaging artifacts were avoided.

As illustrated in Figure 1, the anatomical ROI locations provide the source regions for radiomic feature extraction, whereas the reported distances quantify dissimilarity between the corresponding radiomic feature vectors rather than spatial separation between the ROIs.

For each patient, the four predefined regions were subsequently represented by their corresponding radiomic feature vectors, denoted as T, M, I, and C. Importantly, the ipsilateral and contralateral reference regions were defined as normal-appearing parenchyma rather than histopathologically confirmed healthy tissue. Their inclusion enabled the radiomic characteristics of the peritumoral microenvironment to be interpreted relative to bilateral within-patient parenchymal variation rather than against an external control population.

All ROIs were manually delineated using 3D Slicer (version 5.8.1) by two board-certified radiologists (D.D. and Ö.Ç., with 18 and 16 years of breast imaging experience, respectively). Initial delineations were performed independently. Following the reproducibility assessment, spatial discrepancies between the independent masks were jointly reviewed by both readers and resolved by consensus. The resulting consensus ROIs were used for the final radiomic feature extraction and subsequent distance analyses.

The four ROI types differed systematically in size and sampling geometry by design. The ipsilateral and contralateral reference regions were circular ROIs of 10 mm diameter, corresponding to an in-plane area of approximately 79 mm^2^, whereas the tumor and peritumoral regions followed lesion-related boundaries and were correspondingly larger. For a lesion of the mean maximum diameter observed in this cohort, a 5 mm outward expansion yields a peritumoral ring several times larger in area than the reference ROIs. This difference is an intrinsic consequence of the region definitions rather than a post hoc analytical choice, and its potential influence is addressed in the feature-composition sensitivity analyses (Section 2.7) and discussed as a limitation in Section 4.6.

Segmentation reproducibility was evaluated in a randomly selected subset of 20 patients. Interobserver reproducibility was assessed using independent segmentations performed by the two radiologists, whereas intraobserver reproducibility was assessed by repeat segmentation performed by the primary radiologist after a two-week interval. Reproducibility was quantified at the radiomic-feature level using a two-way mixed-effects, absolute-agreement, single-measure intraclass correlation coefficient [ICC(3,1)]. An ICC threshold of 0.75 was prespecified to indicate good-to-excellent feature reproducibility, and the features retained in the final analysis met this criterion in both the intraobserver and interobserver assessments. This evaluation was performed on the same single-slice ROI representation used for the main analysis and in a subset of 20 patients from a single institution. Because the reference parenchymal ROIs are small, fixed-size regions placed within visually homogeneous fibroglandular tissue, agreement for these regions is expected to be influenced by the constrained ROI geometry as well as by reader agreement on tissue selection. The reported reproducibility should therefore be regarded as an internal consistency check under the present protocol rather than as evidence of general feature stability.

### 2.3. Radiomic Feature Extraction and Preprocessing

Radiomic features were extracted independently from the four predefined tissue regions used in the present analysis: the tumor core (T), peritumoral microenvironment (M), ipsilateral normal-appearing breast parenchyma (I), and contralateral normal-appearing breast parenchyma (C). Feature extraction was performed using PyRadiomics (version 3.1.0.post2+g146e3bd) in Python 3.9.10  [25]. The computational environment additionally included NumPy 1.26.4, SimpleITK 2.4.0rc2.dev213, and PyWavelets 1.6.0.

Radiomic features were extracted from the original, non-subtracted images of the third post-contrast phase of the dynamic contrast-enhanced T1-weighted MRI series. Although subtraction images from the same third post-contrast phase were used for ROI delineation, quantitative feature extraction was performed from the corresponding original DCE-T1-weighted images. As described in Section 2.2, the masks supplied for radiomic feature extraction comprised a single axial slice for each predefined tissue region. The present analysis should therefore be interpreted as a single-slice radiomic analysis rather than a volumetric three-dimensional radiomic analysis.

A fixed gray-level discretization bin width of 25 was used during feature extraction. No image-level intensity normalization, outlier removal, resegmentation, or spatial resampling was applied prior to feature calculation. The original voxel spacing of each MRI examination was therefore preserved. Because all four ROIs for a given patient were extracted from the same bilateral DCE-MRI examination and the same post-contrast phase, the regions entering each within-patient distance calculation shared the same examination-specific spatial sampling and signal-intensity environment. Nevertheless, retaining native voxel spacing may influence radiomic features through differences in physical sampling scale and texture-neighborhood representation across examinations, while the absence of image-level intensity normalization may leave first-order and texture features sensitive to examination-specific signal scaling and enhancement characteristics. The PyRadiomics configuration used a GLCM neighborhood distance of 1 and symmetrical GLCM computation. Although the software configuration retained force2D=False, all masks supplied to the feature-extraction pipeline occupied a single axial slice; consequently, all analyzed radiomic information was derived from the corresponding single-slice ROI representation. Only features derived from the original image type were included, and no additional filtered image transformations, such as wavelet or Laplacian-of-Gaussian filtering, were applied.

The initial PyRadiomics output contained 107 quantitative imaging features, comprising 14 shape features, 18 first-order statistical features, 24 gray-level co-occurrence matrix (GLCM) features, 16 gray-level run-length matrix (GLRLM) features, 16 gray-level size-zone matrix (GLSZM) features, 14 gray-level dependence matrix (GLDM) features, and 5 neighboring gray-tone difference matrix (NGTDM) features. Because all masks supplied to the extraction pipeline occupied a single axial slice, the PyRadiomics shape features were computed from regions with a one-voxel through-plane extent. These descriptors therefore characterize the in-plane geometry of the analyzed cross-section rather than true volumetric lesion morphology, and several of them (for example sphericity, flatness, and surface-to-volume ratio) do not carry their conventional three-dimensional interpretation in this setting. This limitation is considered explicitly in the shape-free sensitivity analysis described in Section 2.7 and in the interpretation of the feature-family results. Feature nomenclature and definitions followed the PyRadiomics implementation; most PyRadiomics feature definitions are aligned with the standardized radiomic feature definitions developed by the Image Biomarker Standardisation Initiative (IBSI), with implementation-specific differences documented by PyRadiomics [26].

Before construction of the analysis matrix, PyRadiomics diagnostic entries were excluded. In addition, a prespecified rule in the analysis pipeline excluded texture features whose PyRadiomics feature names ended with “Normalized”. This rule resulted in the exclusion of five features: GrayLevelNonUniformityNormalized and RunLengthNonUniformityNormalized from the GLRLM family; GrayLevelNonUniformityNormalized and SizeZoneNonUniformityNormalized from the GLSZM family; and DependenceNonUniformityNormalized from the GLDM family. The purpose of this rule was to avoid simultaneously representing closely related absolute and normalized formulations of the same non-uniformity constructs in the primary distance-based feature space. Thus, the rule represented a pipeline-level redundancy-control convention rather than a biological assumption that the non-normalized formulations were intrinsically preferable. These exclusions were rule-based and were applied independently of the observed feature values or study outcomes; they did not constitute data-driven feature selection. Because the retained non-normalized counterparts may be more sensitive to matrix size and ROI voxel count than their normalized variants, we recognized that this choice could potentially influence the calculated radiomic distances. This possibility was therefore evaluated explicitly in a dedicated feature-composition sensitivity analysis, in which the five retained non-normalized measures were replaced by their corresponding normalized PyRadiomics variants (Section 2.7). No additional correlation-based feature selection or dimensionality reduction was applied at this stage.

Following this preprocessing step, 102 radiomic features remained. During subsequent quality control, original__shape__maximum2ddiameterslice and original__shape__ maximum3ddiameter were found to contain identical values across all 224 ROI observations. Review of the PyRadiomics mask diagnostics confirmed that all masks used for feature extraction had a one-voxel extent in the through-plane direction, consistent with the single-slice extraction protocol and explaining the equivalence of these two diameter measures in the analyzed dataset. To avoid redundant weighting of equivalent information in the distance calculations, original__shape__maximum2ddiameterslice was removed and original__shape__maximum3ddiameter was retained. Here, “Maximum3DDiameter” is retained as the PyRadiomics feature name; because the masks have a one-voxel through-plane extent, its value should not be interpreted as a volumetric three-dimensional diameter. The final radiomic feature set therefore consisted of 101 features.

The final feature matrix was systematically screened for missing values, infinite values, duplicated rows, duplicated column names, and zero-variance features. No missing or infinite values were detected, no duplicated ROI rows or duplicated feature names were present, and none of the retained features exhibited zero variance. No formal near-zero-variance threshold was applied, as all retained features showed sufficient variability across the analysis dataset.

For the 56 patients included in the study, the four predefined tissue regions yielded a total of 224 ROI-level observations. Because radiomic features differed substantially in their numerical scales, feature-wise z-score standardization was performed before distance computation using the StandardScaler implementation. A single standardization transformation was fitted across the complete 224×101 ROI-feature matrix and applied consistently to the tumor, peritumoral, ipsilateral, and contralateral feature vectors.

For each feature *j*, the standardized value was calculated as

(1) $$z_{ij}=\frac{x_{ij}-\mu_{j}}{\sigma_{j}},$$

where $x_{ij}$ denotes the original value of feature *j* for ROI observation *i*, and $\mu_{j}$ and $\sigma_{j}$ denote the mean and standard deviation of the corresponding feature across the ROI-level analysis dataset. This standardization step was performed after radiomic feature extraction and therefore did not alter the underlying MRI data.

All subsequent radiomic distance calculations and dimensionality-reduction analyses were performed using the resulting 101-dimensional standardized feature vectors.

### 2.4. Radiomic Distance Framework

Following feature preprocessing and standardization, each tissue region was represented as a 101-dimensional radiomic feature vector. For patient *i*, the standardized feature vectors corresponding to the tumor core, peritumoral microenvironment, ipsilateral normal-appearing breast parenchyma, and contralateral normal-appearing breast parenchyma were denoted as $T_{i}$, $M_{i}$, $I_{i}$, and $C_{i}$, respectively, where $T_{i},M_{i},I_{i},C_{i}\in R^{101}$.

To characterize the radiomic position of the peritumoral microenvironment within the patient-specific tissue context, four pairwise distances were calculated separately for each patient:

(2) $$d_{MT,i}=D\left(M_{i},T_{i}\right),$$

(3) $$d_{MI,i}=D\left(M_{i},I_{i}\right),$$

(4) $$d_{MC,i}=D\left(M_{i},C_{i}\right),$$

and

(5) $$d_{IC,i}=D\left(I_{i},C_{i}\right),$$

where D(·,·) denotes the selected radiomic distance metric.

The distance $d_{MT}$ quantified the radiomic separation between the peritumoral microenvironment and the tumor core. In contrast, $d_{MI}$ and $d_{MC}$ quantified the radiomic separation of the peritumoral microenvironment from ipsilateral and contralateral normal-appearing breast parenchyma, respectively. The bilateral normal-parenchyma distance, $d_{IC}$, was used as a patient-specific internal reference representing the radiomic distance between the selected normal-appearing regions of the two breasts.

Accordingly, the primary analytical question was not whether the peritumoral microenvironment was globally more similar to tumor tissue or normal-appearing tissue, but whether its distance from normal-appearing breast parenchyma exceeded the within-patient bilateral normal-appearing parenchymal reference distance. The two primary comparisons were therefore

(6) $$d_{MI}>d_{IC}$$

and

(7) $$d_{MC}>d_{IC}.$$

The $d_{MT}$ distance was retained as a complementary measure to provide contextual information regarding the radiomic relationship between the tumor core and its surrounding microenvironment, rather than as the primary inferential endpoint.

To evaluate whether the observed relationships were dependent on a specific geometric definition of similarity, four complementary distance measures were applied: Euclidean distance in the standardized feature space, Manhattan distance, cosine distance, and Mahalanobis distance.

For two standardized radiomic vectors x and y with p=101 features, Euclidean distance in the standardized feature space was defined as

(8) $$D_{E}\left(x,y\right)=\sqrt{\sum_{j=1}^{p}{\left(x_{j}-y_{j}\right)}^{2}}.$$

Because all features had been standardized before distance computation, this metric represents Euclidean separation after feature-wise scale normalization and is hereafter referred to as standardized Euclidean distance.

Manhattan distance, representing the cumulative absolute feature-wise difference between two tissue regions, was calculated as

(9) $$D_{L1}\left(x,y\right)=\sum_{j=1}^{p}\left|x_{j}-y_{j}\right|.$$

Cosine distance was used to quantify differences in the orientation of the radiomic feature profiles within the standardized feature space and was defined as

(10) $$D_{\cos}\left(x,y\right)=1-\frac{x^{T}y}{{∥x∥}_{2}{∥y∥}_{2}}.$$

Because feature-wise standardization centers each feature at the cohort mean, the standardized vectors are expressed relative to the mean ROI profile. In this setting cosine distance reflects the angular relationship between mean-centered profiles and is therefore not strictly independent of overall feature magnitude. Finally, Mahalanobis distance was used to account for the covariance structure among radiomic features:

(11) $$D_{\mathrm{Mah}}\left(x,y\right)=\sqrt{{\left(x-y\right)}^{T}\Sigma^{-1}\left(x-y\right)},$$

where Σ denotes the covariance matrix used to characterize the reference radiomic feature space. Because the number of radiomic features exceeded the number of available contralateral reference observations, covariance estimation required regularization. The estimation of Σ and the corresponding Mahalanobis sensitivity analyses are described separately in Section 2.5.

In addition to the four primary distance quantities, a microenvironment similarity index (MSI) was calculated as a secondary descriptive measure:

(12) $${\mathrm{MSI}}_{i}=d_{MI,i}-d_{MT,i}.$$

A positive MSI indicates that the peritumoral microenvironment is radiomically closer to the tumor core than to ipsilateral normal-appearing parenchyma, whereas a negative MSI indicates greater proximity to the ipsilateral normal-appearing parenchyma. Because MSI directly compares tumor- and normal-oriented distances, it was treated as a descriptive contextual index and was not used as the primary inferential endpoint.

Across all distance metrics, larger distance values indicate greater radiomic dissimilarity between the corresponding tissue regions. All comparisons were calculated within individual patients, thereby preserving the paired intra-patient design throughout the analysis.

### 2.5. Mahalanobis Covariance Estimation and Sensitivity Analysis

Unlike Euclidean, Manhattan, and cosine distances, Mahalanobis distance incorporates the covariance structure among radiomic features. In the present study, feature-wise standardization was first performed using the complete 224-ROI analysis matrix, as described in Section 2.3. After this global standardization step, the covariance matrix used for Mahalanobis distance calculation was estimated exclusively from the 56 contralateral normal-appearing breast parenchymal feature vectors. The contralateral normal-appearing compartment was used as the covariance reference so that the Mahalanobis geometry would be defined from a single, consistently sampled normal-appearing tissue compartment rather than from a mixture of tumor, peritumoral, and reference regions. Including tumor or peritumoral observations in the covariance estimate would incorporate variation from the very regions whose deviation was being quantified, whereas pooling anatomically distinct ROI types would make the covariance structure dependent on their relative representation. The contralateral region was therefore selected as a common reference compartment because it was spatially separated from the index tumor and had the same predefined role across all patients. This choice does not imply that contralateral parenchyma represents histologically normal tissue or a physiological gold standard; it serves only as the reference population used to define covariance in the Mahalanobis feature space. Thus, although the scaling parameters were derived from the complete analysis dataset, the covariance structure itself was determined only from the contralateral normal-appearing reference observations.

The resulting reference matrix consisted of 56 contralateral normal-appearing parenchymal observations represented in the final 101-dimensional standardized radiomic feature space. Because the number of features exceeded the number of available reference observations (p=101, n=56), the empirical sample covariance matrix was necessarily rank-deficient, with a maximum possible rank of n−1=55, and could not be reliably inverted using the conventional matrix inverse.

To obtain a stable and invertible covariance estimate for the primary Mahalanobis analysis, Ledoit–Wolf shrinkage covariance estimation was employed [27]. This approach regularizes the empirical covariance matrix by shrinking it toward a structured, scaled-identity target, thereby reducing instability associated with high-dimensional covariance estimation.

Conceptually, the shrinkage covariance estimator can be expressed as

(13) $${\hat{\Sigma }}_{\mathrm{LW}}=\left(1-\delta \right)S+\delta \mu I,$$

where S denotes the empirical covariance matrix of the contralateral normal-appearing parenchymal feature vectors, I is the p×p identity matrix,

(14) $$\mu =\frac{\mathrm{tr}(S)}{p},$$

and δ represents the shrinkage intensity. For the primary Mahalanobis analysis, the shrinkage intensity was estimated automatically using the Ledoit–Wolf procedure rather than being specified a priori. The resulting regularized precision matrix, ${\hat{\Sigma }}_{\mathrm{LW}}^{-1}$, was subsequently used in the Mahalanobis distance calculation.

Accordingly, the primary Mahalanobis distance between two standardized radiomic feature vectors x and y was calculated as

(15) $$D_{\mathrm{Mah},\mathrm{LW}}\left(x,y\right)=\sqrt{{\left(x-y\right)}^{T}{\hat{\Sigma }}_{\mathrm{LW}}^{-1}\left(x-y\right)}.$$

Using this common covariance model, the four patient-specific Mahalanobis distances $d_{MT}$, $d_{MI}$, $d_{MC}$, and $d_{IC}$ were recalculated for all 56 patients. The automatically estimated shrinkage coefficient and the condition numbers of the empirical and Ledoit–Wolf covariance matrices were recorded as numerical diagnostics of covariance regularization.

To assess whether the principal findings depended on the choice of covariance estimator, two complementary sensitivity analyses were performed. First, the empirical covariance matrix was retained and its Moore–Penrose pseudo-inverse was used in place of a conventional inverse. This approach corresponded to the covariance treatment used in the initial analysis and was retained only as a robustness reference rather than as the primary Mahalanobis estimator.

Second, a fixed-shrinkage sensitivity analysis was conducted over a broad range of shrinkage intensities. For δ∈{0.05,0.10,0.25,0.50,0.75,1.00}, the covariance matrix was calculated as

(16) $${\hat{\Sigma }}_{\delta }=\left(1-\delta \right)S+\delta \left(\frac{\mathrm{tr}(S)}{p}\right)I,$$

and the corresponding inverse covariance matrix was used to recalculate all patient-specific Mahalanobis distances. At δ=1, the covariance estimate reduces to a scaled identity matrix; consequently, the resulting Mahalanobis distance is equivalent to Euclidean distance in the standardized feature space up to a constant scaling factor.

For each covariance estimator, the two principal comparisons, $d_{MI}$ versus $d_{IC}$ and $d_{MC}$ versus $d_{IC}$, were repeated using the same paired statistical framework described in Section 2.6. The direction and magnitude of the corresponding effects were examined across covariance estimators to determine whether the primary conclusions were robust to covariance regularization.

Finally, patient-level agreement between the Mahalanobis distances obtained using the Moore–Penrose pseudo-inverse and those obtained using the primary Ledoit–Wolf estimator was assessed using Spearman rank correlations separately for $d_{MT}$, $d_{MI}$, $d_{MC}$, and $d_{IC}$.

### 2.6. Statistical Analysis

All statistical comparisons were performed at the patient level while preserving the paired intra-patient structure of the study. For each of the four radiomic distance metrics, descriptive statistics were calculated for $d_{MT}$, $d_{MI}$, $d_{MC}$, $d_{IC}$, and the microenvironment similarity index (MSI). Continuous distance measures were summarized using the mean and standard deviation.

The primary statistical analyses were designed to determine whether the peritumoral-to-normal radiomic distances exceeded the within-patient bilateral normal-appearing parenchymal reference distance. Accordingly, two paired differences were defined for each distance metric:

(17) $$\Delta_{MI,i}=d_{MI,i}-d_{IC,i},$$

and

(18) $$\Delta_{MC,i}=d_{MC,i}-d_{IC,i}.$$

Positive values of $\Delta_{MI}$ or $\Delta_{MC}$ therefore indicate that the corresponding peritumoral-to-normal radiomic distance exceeds $d_{IC}$ within that patient. For consistency with the non-parametric primary analysis, the patient-level paired differences were additionally summarized using the median and interquartile range, reported as the 25th and 75th percentiles (Q1–Q3).

To provide a common and distribution-robust inferential framework across all distance metrics, two-sided Wilcoxon signed-rank tests were used as the primary statistical tests. Although the expected direction of the effect was $d_{MI}>d_{IC}$ and $d_{MC}>d_{IC}$, two-sided tests were used to allow departures in either direction and to provide a conservative inferential assessment.

For each Wilcoxon comparison, the number of patients with positive and negative paired differences was recorded. Effect magnitude was quantified using the matched-pairs rank-biserial correlation,

(19) $$r_{\mathrm{rb}}=\frac{R_{+}-R_{-}}{R_{+}+R_{-}},$$

where $R_{+}$ and $R_{-}$ denote the sums of the signed ranks corresponding to positive and negative paired differences, respectively. Confidence intervals for $r_{\mathrm{rb}}$ were estimated using 10,000 patient-level bootstrap resamples with replacement. The 2.5th and 97.5th percentiles of the bootstrap distribution were used to construct 95% confidence intervals. A fixed random seed of 42 was used to ensure reproducibility of the bootstrap procedure.

The two primary comparisons were evaluated separately using standardized Euclidean, Manhattan, cosine, and Ledoit–Wolf Mahalanobis distances, resulting in a total of eight primary hypothesis tests. To control the false discovery rate associated with these comparisons, the eight Wilcoxon *p*-values were adjusted jointly using the Benjamini–Hochberg procedure [28]. Statistical significance for the primary analyses was defined using a false-discovery-rate-adjusted *q*-value of <0.05. The unadjusted Wilcoxon *p*-values were used as input to the Benjamini–Hochberg procedure, and the resulting adjusted *q*-values were used for primary statistical inference and reported for the primary comparisons.

As a complementary parametric sensitivity analysis, paired Student’s *t*-tests were also performed for each of the eight primary comparisons. For each comparison, the mean paired difference and its 95% confidence interval were calculated. Standardized paired effect sizes were expressed using Cohen’s $d_{z}$:

(20) $$d_{z}=\frac{\bar{\Delta }}{s_{\Delta }},$$

where $\bar{\Delta }$ and $s_{\Delta }$ denote the mean and standard deviation of the corresponding within-patient differences, respectively. The paired *t*-test analyses were considered supportive sensitivity analyses and were not used as the primary basis for statistical inference. Detailed parametric sensitivity results are provided in Appendix A.

The robustness of Mahalanobis-based findings to covariance estimation was evaluated using the alternative covariance formulations described in Section 2.5. For each alternative estimator, the direction and magnitude of the paired differences, Wilcoxon signed-rank results, matched-pairs rank-biserial correlations, and numbers of patients with positive paired differences were compared with those obtained using the primary Ledoit–Wolf estimator. These analyses were interpreted as robustness assessments rather than as additional independent primary hypotheses. Detailed results of the alternative covariance formulations are provided in Appendix B.

As a complementary reduced-dimensional sensitivity analysis, principal component analysis (PCA) was applied to the standardized 101-feature matrix, and the distance analyses were repeated using the first two, three, and four principal components. In each PCA space, Mahalanobis covariance was estimated exclusively from the contralateral normal-appearing parenchymal observations. Because the resulting reduced-dimensional covariance matrices were full rank, conventional matrix inversion was used. The PCA-based paired comparisons were considered exploratory and were not included in the eight-test primary multiple-comparison family. Accordingly, the Wilcoxon *p*-values from these PCA sensitivity analyses were reported without additional multiple-comparison adjustment. Detailed results are provided in Appendix C.

All statistical analyses were conducted using Python 3.9.10 with the scientific computing and statistical libraries used in the finalized analysis pipeline. All analyses were based on the complete paired dataset of 56 patients, with no imputation required for the radiomic feature matrix.

### 2.7. Feature-Composition Sensitivity Analyses

Additional sensitivity analyses were performed to assess whether the principal findings were disproportionately influenced by explicit shape information or by texture features that may be sensitive to ROI size. These analyses were conducted as complementary robustness assessments and did not replace the primary 101-feature analysis.

First, a shape-free sensitivity analysis was performed after excluding all 13 shape features retained in the final 101-feature dataset. The resulting 88-feature space therefore contained only first-order and texture features. Feature-wise standardization was refitted across all 224 ROI observations within this reduced feature space, after which standardized Euclidean, Manhattan, cosine, and Ledoit–Wolf Mahalanobis distances were recalculated. The same paired comparisons, $d_{MI}-d_{IC}$ and $d_{MC}-d_{IC}$, were then evaluated using the statistical framework described in Section 2.6.

Second, separate feature-family sensitivity analyses were performed for shape (13 features), first-order (18 features), GLCM (24 features), GLRLM (14 features), GLSZM (14 features), GLDM (13 features), and NGTDM (5 features). Each feature family was independently standardized and treated as a separate radiomic feature space. Within each feature family, the same four distance metrics and two paired comparisons were evaluated to determine whether the observed peritumoral deviation was concentrated within a particular category of radiomic features.

Third, an additional shape-free sensitivity analysis was performed to assess the potential influence of non-normalized non-uniformity measures. This analysis was specifically designed to determine whether the primary feature-exclusion rule could have systematically favored ROI-size-sensitive non-normalized measures and thereby influenced the principal distance comparisons. Five non-normalized texture features retained in the primary pipeline were replaced by their corresponding normalized PyRadiomics variants: GrayLevelNonUniformityNormalized and RunLengthNonUniformityNormalized from GLRLM, GrayLevelNonUniformityNormalized and SizeZoneNonUniformityNormalized from GLSZM, and DependenceNonUniformityNormalized from GLDM. The resulting feature space again contained 88 features. Feature-wise standardization was refitted across all 224 ROI observations, and the four radiomic distance metrics and two primary paired comparisons were recalculated using the same analytical procedure.

For each sensitivity analysis, the direction of the paired differences, Wilcoxon signed-rank statistics, matched-pairs rank-biserial correlations with 95% bootstrap confidence intervals, numbers of patients with positive paired differences, and complementary paired *t*-test effect sizes were evaluated. Benjamini–Hochberg correction was applied separately to the eight paired comparisons within each evaluated sensitivity-analysis feature space. These analyses were interpreted as robustness assessments and were not included in the multiple-comparison family of the primary 101-feature analysis. Detailed results are provided in Appendix D. These analyses address the contribution of explicit shape descriptors and of selected size-sensitive non-uniformity measures. They do not constitute a formal size-matched comparison, and residual dependence on ROI area, voxel count, or texture-matrix stability therefore cannot be excluded on the basis of the present analyses.

## 3. Results

The final analysis comprised 56 patients, with four predefined tissue regions evaluated for each patient in a 101-dimensional standardized radiomic feature space. Radiomic relationships among the tumor core, peritumoral microenvironment, ipsilateral normal-appearing parenchyma, and contralateral normal-appearing parenchyma were assessed using standardized Euclidean, Manhattan, cosine, and Ledoit–Wolf Mahalanobis distances. Across all four metrics, the peritumoral-to-normal distances were consistently greater than the bilateral distance between normal-appearing parenchymal regions. Both primary comparisons, $d_{MI}$ versus $d_{IC}$ and $d_{MC}$ versus $d_{IC}$, remained statistically significant after correction for multiple testing, with consistently large paired effect sizes. The following subsections present the descriptive distance distributions, primary paired comparisons, and covariance-sensitivity analyses in detail. The ROI configuration and the definitions of the principal pairwise radiomic distances are illustrated schematically in Figure 1.

### 3.1. Study Cohort and Final Radiomic Dataset

A total of 56 patients were included in the final analysis. For each patient, four predefined tissue regions were available—the tumor core (T), peritumoral microenvironment (M), ipsilateral normal-appearing breast parenchyma (I), and contralateral normal-appearing breast parenchyma (C)—resulting in 224 ROI-level observations.

Following the radiomic preprocessing and quality-control procedures described in Section 2.3, the final feature space comprised 101 radiomic features. No patients or ROI observations were excluded because of missing or non-finite radiomic feature values. All subsequent full-space distance analyses were therefore performed using the complete 224×101 standardized radiomic feature matrix.

The mean patient age was 53.75 ± 13.95 years, with a median age of 52.5 years and a range of 30–85 years. Postoperative histopathological data were available for 47 of the 56 patients. Among these patients, invasive ductal/NOS carcinoma was the most common pathological diagnosis (31/47, 66.0%), followed by ductal carcinoma in situ/in situ carcinoma (8/47, 17.0%), invasive lobular carcinoma (4/47, 8.5%), and other special invasive carcinoma types (4/47, 8.5%). The latter group comprised invasive carcinoma with cribriform–papillary features, invasive carcinoma with focal mucinous differentiation, invasive solid papillary carcinoma, and medullary carcinoma.

Hormone-receptor results were available for 46 patients. Estrogen-receptor positivity was observed in 40/46 patients (87.0%), whereas progesterone-receptor positivity was observed in 38/46 patients (82.6%). Ki-67 measurements were available for 45 patients, with a mean index of 23.56 ± 22.76% and a median of 15%. The mean maximum tumor diameter measured on MRI was 24.5 ± 12.3 mm.

Because postoperative clinicopathological information was not uniformly available for all patients, incomplete variables were not summarized as if they represented the entire cohort. In particular, distributions of variables for which complete or sufficiently verified data were unavailable were not reported.

Of the 112 patients assessed for eligibility during the predefined study period, 56 were excluded according to the prespecified criteria and the remaining 56 eligible patients were consecutively included in the final analysis, as detailed in Section 2.1.

### 3.2. Radiomic Distance Distributions

**Figure 2:**
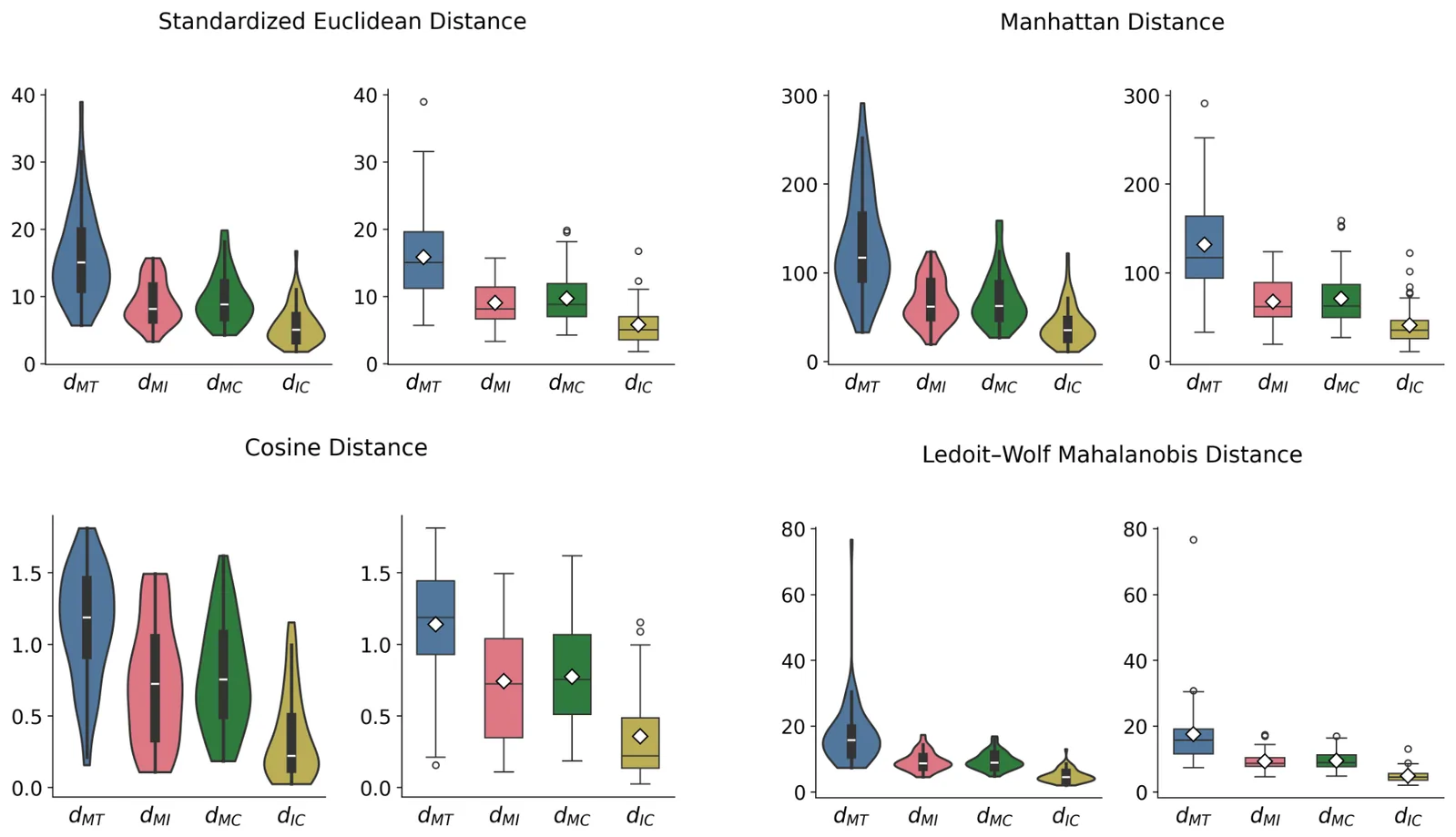
Distribution of patient-specific radiomic distances in the final 101-feature dataset across the four distance metrics: standardized Euclidean (**top left**), Manhattan (**top right**), cosine (**bottom left**), and Ledoit–Wolf Mahalanobis (**bottom right**). For each metric, violin and box plots summarize the distributions of $d_{MT}$, $d_{MI}$, $d_{MC}$, and $d_{IC}$ across 56 patients. Across all metrics, $d_{IC}$ was concentrated at lower values than $d_{MI}$ and $d_{MC}$, consistent with greater peritumoral-to-normal radiomic deviation than within-patient bilateral normal-appearing parenchymal reference distance.

Descriptive statistics of the patient-specific radiomic distances are presented in Table 1, and the corresponding distance distributions are visualized in Figure 2. A consistent pattern was observed across all four distance metrics. Based on the group means, the largest separation was observed between the peritumoral microenvironment and the tumor core ($d_{MT}$), whereas the smallest separation was consistently observed between ipsilateral and contralateral normal-appearing breast parenchyma ($d_{IC}$). The peritumoral-to-normal distances ($d_{MI}$ and $d_{MC}$) occupied an intermediate position, with the same mean ordering across all metrics: $d_{MT}>d_{MC}>d_{MI}>d_{IC}$.

For standardized Euclidean distance, mean ± standard deviation values were 15.84±6.65 for $d_{MT}$, 9.06±3.25 for $d_{MI}$, 9.67±3.74 for $d_{MC}$, and 5.76±2.90 for $d_{IC}$. Manhattan distance showed a comparable pattern, with mean values of 131.67±57.52, 67.17±25.46, 70.60±30.39, and 40.78±22.35, respectively.

For cosine distance, the corresponding mean values were 1.139±0.390 for $d_{MT}$, 0.742±0.417 for $d_{MI}$, 0.775±0.367 for $d_{MC}$, and 0.357±0.300 for $d_{IC}$. Using the primary Ledoit–Wolf Mahalanobis formulation, mean distances were 17.52±9.98, 9.29±2.84, 9.47±2.63, and 4.91±2.00 for $d_{MT}$, $d_{MI}$, $d_{MC}$, and $d_{IC}$, respectively.

Using ratios of group means, $d_{MI}$ was 1.57, 1.65, 2.07, and 1.89 times $d_{IC}$ for standardized Euclidean, Manhattan, cosine, and Mahalanobis distances, respectively. The corresponding $d_{MC}/d_{IC}$ ratios were 1.68, 1.73, 2.17, and 1.93. These descriptive findings consistently indicated greater peritumoral-to-normal radiomic separation than the bilateral normal-appearing parenchymal reference distance.

The MSI showed negative mean values for all four metrics (−6.78 for standardized Euclidean, −64.51 for Manhattan, −0.397 for cosine, and −8.23 for Mahalanobis distance). Thus, although the peritumoral region exhibited greater separation from normal-appearing parenchyma than the bilateral normal-appearing parenchymal reference distance, its absolute radiomic distance to the tumor core was, on average, greater than its distance to the ipsilateral normal-appearing parenchyma. This observation was treated as descriptive and was not part of the primary hypothesis testing.

Figure 2 visually confirms the descriptive pattern observed in Table 1. Across all four distance metrics, the bilateral normal-appearing parenchymal distance ($d_{IC}$) was concentrated at lower values than the peritumoral-to-normal distances ($d_{MI}$ and $d_{MC}$). In contrast, $d_{MT}$ generally occupied the highest range of values. Although partial overlap between the distributions was present, the overall ordering was preserved consistently across Euclidean, Manhattan, cosine, and Mahalanobis formulations.

### 3.3. Peritumoral Deviation Beyond the Bilateral Normal-Appearing Parenchymal Reference

The primary paired analyses consistently showed that the radiomic distance between the peritumoral microenvironment and normal-appearing breast parenchyma exceeded the bilateral distance between ipsilateral and contralateral normal-appearing parenchymal regions. All eight primary comparisons remained statistically significant after Benjamini–Hochberg correction for multiple testing (Table 2).

Consistent with the Wilcoxon-based inference, the median paired differences were positive for all eight primary comparisons. Moreover, the first quartile (Q1) was above zero in every comparison, indicating that the positive direction was also evident across the central portions of the paired-difference distributions rather than being driven solely by large positive observations. Complementary paired *t*-test analyses yielded results consistent with the primary Wilcoxon findings; detailed parametric sensitivity statistics are provided in Appendix A.

For standardized Euclidean distance, $d_{MI}$ exceeded $d_{IC}$ by a mean paired difference of 3.29 (Wilcoxon W=179, $q=5.07\times 10^{-7}$), with a matched-pairs rank-biserial correlation of $r_{\mathrm{rb}}=0.776$ (95% CI: 0.581–0.926). A positive difference was observed in 47 of 56 patients. The corresponding $d_{MC}-d_{IC}$ comparison showed a mean difference of 3.91 (W=78, $q=1.14\times 10^{-8}$), with $r_{\mathrm{rb}}=0.902$ (95% CI: 0.777–0.984) and positive differences in 50 of 56 patients.

The same pattern was observed using Manhattan distance. The mean $d_{MI}-d_{IC}$ difference was 26.39 (W=178, $q=5.07\times 10^{-7}$), with $r_{\mathrm{rb}}=0.777$ (95% CI: 0.580–0.924), while the mean $d_{MC}-d_{IC}$ difference was 29.82 (W=117, $q=4.44\times 10^{-8}$), with $r_{\mathrm{rb}}=0.853$ (95% CI: 0.702–0.961). Positive paired differences were observed in 46 and 49 patients, respectively.

For cosine distance, $d_{MI}-d_{IC}$ had a mean paired difference of 0.384 (W=200, $q=1.07\times 10^{-6}$), with $r_{\mathrm{rb}}=0.749$ (95% CI: 0.541–0.909) and positive differences in 46 of 56 patients. The $d_{MC}-d_{IC}$ comparison yielded a mean difference of 0.417 (W=107, $q=3.47\times 10^{-8}$), with $r_{\mathrm{rb}}=0.866$ (95% CI: 0.718–0.972), and was positive in 50 of 56 patients.

The strongest paired effects were observed using the primary Ledoit–Wolf Mahalanobis distance. The mean $d_{MI}-d_{IC}$ difference was 4.38 (W=44, $q=3.09\times 10^{-9}$), with $r_{\mathrm{rb}}=0.945$ (95% CI: 0.851–1.000), and 53 of 56 patients showed a positive difference. For $d_{MC}-d_{IC}$, the mean paired difference was 4.56 (W=9, $q=9.82\times 10^{-10}$), with $r_{\mathrm{rb}}=0.989$ (95% CI: 0.957–1.000); this relationship was observed in 55 of 56 patients.

Overall, the direction of the effect was consistent across distance metrics and across individual patients. Within the present cohort and under the ROI definitions and feature space described above, the peritumoral-to-normal radiomic distances therefore exceeded the within-patient bilateral normal-appearing parenchymal reference distance.

### 3.4. Mahalanobis Covariance Sensitivity Analysis

The empirical covariance matrix estimated from the 56 contralateral normal-appearing parenchymal observations was rank-deficient in the 101-dimensional feature space, with a rank of 55/101 and a condition number of $4.61\times 10^{18}$. Ledoit–Wolf regularization substantially improved numerical stability, reducing the covariance condition number to 173.65. The automatically estimated shrinkage intensity was δ=0.2082.

Using the primary Ledoit–Wolf Mahalanobis formulation, the mean paired difference for $d_{MI}-d_{IC}$ was 4.38, with a rank-biserial correlation of $r_{\mathrm{rb}}=0.945$ and a positive difference in 53 of 56 patients. For $d_{MC}-d_{IC}$, the corresponding mean paired difference was 4.56, with $r_{\mathrm{rb}}=0.989$ and positive differences in 55 of 56 patients. The Benjamini–Hochberg-adjusted results for these primary comparisons are reported in Table 2.

The same direction of effect was preserved when the empirical covariance matrix was evaluated using the Moore–Penrose pseudo-inverse. Under this formulation, positive differences were observed in 55 of 56 patients for both primary comparisons, with rank-biserial correlations of 0.996 for $d_{MI}-d_{IC}$ and 0.994 for $d_{MC}-d_{IC}$.

Fixed-shrinkage sensitivity analyses further confirmed the robustness of the findings. Across shrinkage intensities ranging from δ=0.05 to δ=1.00, both $d_{MI}-d_{IC}$ and $d_{MC}-d_{IC}$ remained positive and statistically significant. Rank-biserial effect sizes ranged from 0.776 to 0.960 for $d_{MI}-d_{IC}$ and from 0.902 to 0.991 for $d_{MC}-d_{IC}$. Detailed results for the Moore–Penrose and fixed-shrinkage sensitivity analyses are provided in Appendix B.

Patient-level rank agreement between the Moore–Penrose and Ledoit–Wolf Mahalanobis distances was highest for $d_{MT}$ (Spearman ρ=0.909), while moderate correlations were observed for $d_{MI}$ (ρ=0.608), $d_{MC}$ (ρ=0.507), and $d_{IC}$ (ρ=0.604). Although the absolute geometry and patient ordering differed between covariance estimators, the direction and inferential conclusions of the primary peritumoral-versus-normal comparisons remained stable.

**Figure A1:**
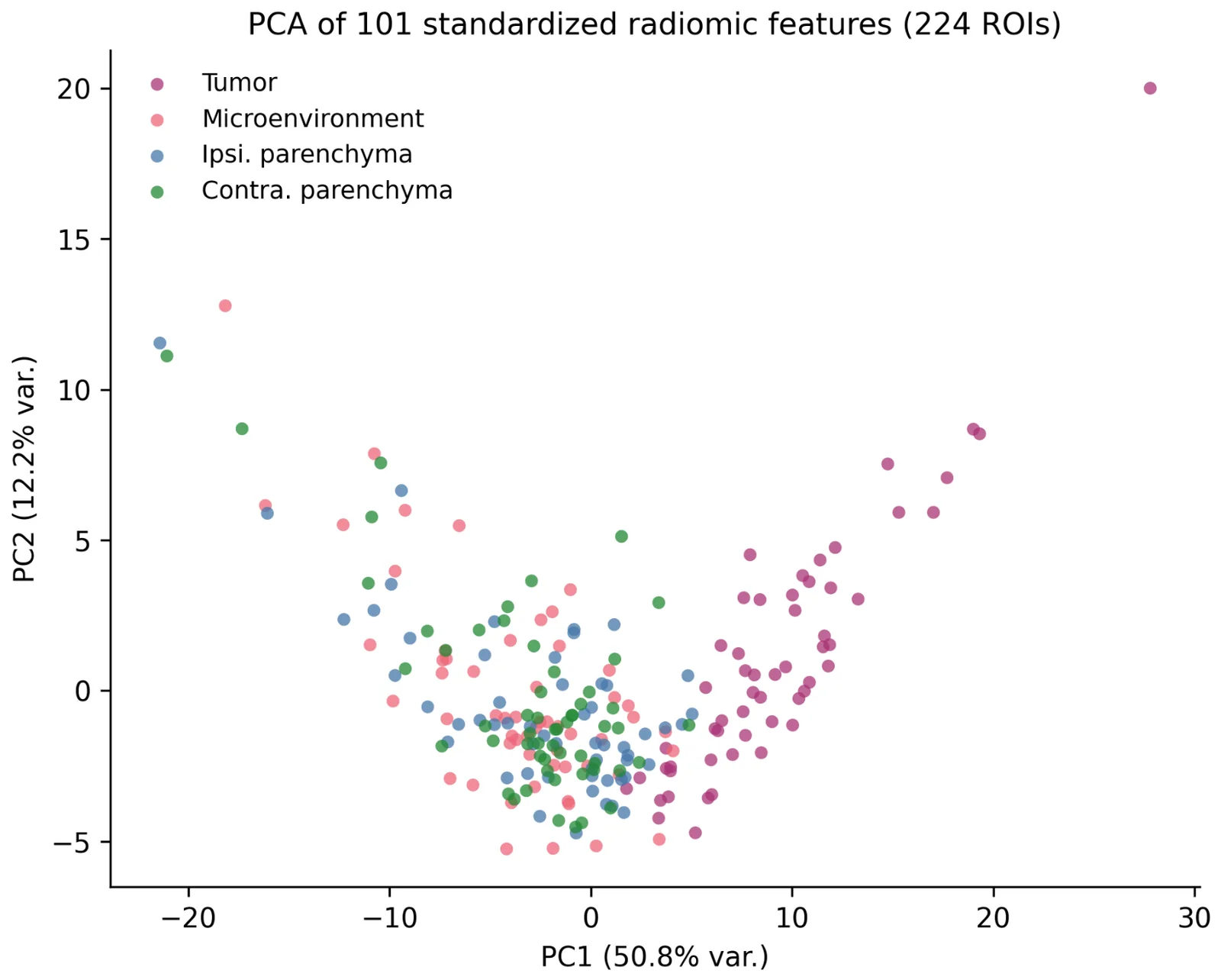
Two-dimensional principal component analysis of the standardized 101-feature radiomic space comprising 224 ROI observations. Each point represents one ROI from the tumor core, peritumoral microenvironment, ipsilateral normal-appearing breast parenchyma, or contralateral normal-appearing breast parenchyma. PC1 and PC2 explained 50.8% and 12.2% of the total variance, respectively. Tumor observations showed a tendency toward separation along PC1, whereas the peritumoral and normal-appearing parenchymal regions demonstrated substantial overlap in the two-dimensional projection.

Complementary reduced-dimensional analyses based on principal component analysis are provided in Appendix C. As shown in Figure A1, tumor observations exhibited a tendency toward separation along the first principal component, whereas the peritumoral and normal-appearing parenchymal regions showed substantial overlap in the two- dimensional projection.

### 3.5. Feature-Composition Sensitivity Analyses

The principal findings remained stable after exclusion of all explicit shape features. Removal of the 13 shape features retained in the primary 101-feature dataset resulted in an 88-feature space containing only first-order and texture features. Across standardized Euclidean, Manhattan, cosine, and Ledoit–Wolf Mahalanobis distances, both $d_{MI}-d_{IC}$ and $d_{MC}-d_{IC}$ remained positive, and all eight paired comparisons remained statistically significant after Benjamini–Hochberg correction, with adjusted *q*-values ranging from $9.8\times 10^{-9}$ to $8.4\times 10^{-6}$. Matched-pairs rank-biserial correlations ranged from 0.68 to 0.93, and positive paired differences were observed in 44–52 of 56 patients, depending on the metric and comparison. Effect sizes were moderately attenuated relative to the primary 101-feature analysis, with Cohen’s $d_{z}$ decreasing by approximately 9.6–24.1%. For the Ledoit–Wolf Mahalanobis distance, $d_{z}$ decreased from 1.44 to 1.09 for $d_{MI}-d_{IC}$ and from 1.70 to 1.29 for $d_{MC}-d_{IC}$. Thus, although explicit shape information contributed to the magnitude of the observed multivariate separation, the principal radiomic relationship persisted after its removal.

Feature-family analyses showed a consistent direction of effect across all evaluated radiomic feature classes. Shape, first-order, GLCM, GLRLM, GLSZM, GLDM, and NGTDM features were analyzed separately, and all 56 evaluated family–metric–comparison combinations yielded positive paired differences and remained statistically significant after the corresponding within-feature-space Benjamini–Hochberg correction. Because these feature-family analyses were exploratory robustness assessments, multiplicity correction was applied within each feature family rather than jointly across all 56 family–metric–comparison combinations. Shape features produced the strongest effects overall, whereas first-order features showed the weakest effects. As noted in Section 2.3, however, the shape descriptors were computed from single-slice masks and therefore reflect differences in the in-plane geometry and extent of the analyzed regions rather than volumetric lesion morphology. The strong shape-family effects are consistent with the fact that the four ROI types differ geometrically by construction and should not be interpreted as an independent biological finding. For this reason, the shape-free analysis reported above is the more informative of the two results. Nevertheless, the expected direction was preserved across every evaluated feature family and distance metric, indicating that the observed peritumoral deviation was not confined to a single category of radiomic features.

The additional shape-free analysis in which five non-normalized non-uniformity features were replaced by their corresponding normalized PyRadiomics variants yielded similar results. Across all four distance metrics, both primary paired comparisons remained positive, and all eight comparisons remained statistically significant after Benjamini–Hochberg correction, with adjusted *q*-values ranging from $1.3\times 10^{-8}$ to $1.5\times 10^{-5}$. Matched-pairs rank-biserial correlations ranged from 0.66 to 0.93, and positive paired differences were observed in 43–52 of 56 patients. Relative to the shape-free analysis, the reduction in Cohen’s $d_{z}$ ranged from approximately 0% to 14.9%, whereas the Mahalanobis effect sizes changed only minimally.

Taken together, these sensitivity analyses indicate that the principal peritumoral-versus-normal distance relationships were not solely attributable to explicit shape features or to the evaluated non-normalized non-uniformity measures. The preservation of the same direction of effect across all evaluated feature families further supports the presence of a broader multivariate radiomic pattern. Detailed sensitivity-analysis results are provided in Appendix D. Given that the excluded shape descriptors do not carry a volumetric interpretation in the present single-slice setting, the 88-feature shape-free results reported in Table A5 may be regarded as the analytically more conservative representation of the principal finding. Both primary comparisons remained positive and statistically significant in that feature space across all four distance metrics.

## 4. Discussion

### 4.1. Principal Findings and Biological Interpretation

The present findings indicate that, under the current ROI definitions, the peritumoral-to-normal radiomic distances consistently exceeded the within-patient bilateral normal-appearing parenchymal reference distance. This pattern was consistent across all four distance formulations and was observed for both ipsilateral and contralateral normal-appearing reference regions. The use of two anatomically distinct normal-appearing tissue references therefore suggests that the observed effect is not restricted to a single comparison with the tumor-bearing breast.

The descriptive relationship between the peritumoral microenvironment and the tumor core should be interpreted separately from the primary peritumoral-to-normal comparisons. Across all four distance metrics, $d_{MT}$ showed the largest mean value and the mean MSI was negative, indicating that, in the present feature space, the peritumoral region was on average more distant from the tumor core than from the ipsilateral normal-appearing parenchyma. However, $d_{MT}$ and MSI were included as secondary descriptive measures and were not used as primary inferential endpoints. Accordingly, these observations should not be interpreted as establishing a specific biological position of the peritumoral tissue along a tumor-to-normal continuum.

The observed radiomic deviation of the peritumoral region may plausibly reflect local differences in enhancement, tissue composition, stromal characteristics, inflammatory processes, edema, or other microenvironment-related alterations. However, these mechanisms were not directly assessed histopathologically in the present study. Therefore, the current findings support a quantitative radiomic distinction between the evaluated tissue regions but do not establish the biological substrate underlying that distinction.

The consistently larger $d_{MI}$ and $d_{MC}$ values relative to $d_{IC}$ indicate that, within the present cohort and analytical framework, the peritumoral-to-normal radiomic distances exceeded the bilateral normal-appearing parenchymal reference distance. This distinction is important because normal-appearing breast parenchyma itself may exhibit substantial inter- and intra-individual heterogeneity. By incorporating $d_{IC}$ as an internal patient-specific reference, the present analysis contextualizes the observed peritumoral deviation relative to the background level of bilateral parenchymal variability.

Across all four distance metrics, the group-level mean $d_{MC}$ values were slightly larger than the corresponding $d_{MI}$ values. However, the present study was not designed to formally compare $d_{MI}$ and $d_{MC}$, and no direct inferential test between these two distances was performed. This numerical difference should therefore be interpreted as descriptive only and should not be considered evidence of a laterality-specific biological effect.

The high proportion of patients showing positive paired differences further supports the consistency of the observed pattern at the individual level rather than only at the level of group averages. Depending on the distance metric, $d_{MI}>d_{IC}$ was observed in 46–53 of 56 patients, while $d_{MC}>d_{IC}$ was observed in 49–55 of 56 patients. This patient-level consistency strengthens the interpretation that the peritumoral radiomic deviation represents a systematic feature of the analyzed cohort rather than an effect driven by a limited number of extreme observations. Nevertheless, the present cohort was not designed or powered to determine whether the magnitude of this deviation differs according to tumor subtype, tumor size, histological grade, molecular characteristics, breast density, or other clinicopathological factors. These relationships should be evaluated in larger cohorts as hypotheses for future investigation.

### 4.2. Comparison with Previous Peritumoral Radiomics Studies

The present findings are consistent with a growing body of breast MRI radiomics literature indicating that the tissue surrounding a breast tumor contains quantitative imaging information that is not captured by analysis of the tumor core alone. Braman et al. demonstrated that peritumoral radiomic features derived from breast DCE-MRI contributed information relevant to treatment response and tumor biology [9]. In a subsequent study of HER2-positive breast cancer, the same group showed that peritumoral features discriminated the HER2-enriched molecular subtype and that combined intratumoral and peritumoral information improved discrimination compared with intratumoral features alone [10]. Notably, features from the immediate peritumoral region were also associated with tumor-infiltrating lymphocyte density, providing evidence that quantitative MRI characteristics surrounding the tumor may reflect aspects of the local tumor microenvironment.

Subsequent studies have extended these observations to additional molecular and biological endpoints. Li et al. reported that radiomic signatures derived from intratumoral and peritumoral regions on functional parametric maps from DCE-MRI were informative for the assessment of HER2 and Ki-67 status [11]. Similarly, Wu et al. demonstrated that both intratumoral and peritumoral MRI radiomics contributed to the evaluation of PD-L1 expression, with the peritumoral signature showing substantial discriminatory performance [12]. More recent multiparametric MRI studies have further shown that the definition and spatial extent of the peritumoral region can influence model performance. Zhou et al. systematically evaluated multiple peritumoral widths and found that the most informative region varied according to MRI sequence, with a 4-mm peritumoral region frequently providing strong predictive information for HER2 status [17]. Cao et al. likewise demonstrated that integration of intratumoral and 3-mm peritumoral features from multiparametric MRI provided robust HER2-status prediction across internal and external validation cohorts [20].

The relevance of peritumoral radiomics has also been demonstrated for prognostic stratification. Chen et al. evaluated multiple peritumoral expansions in patients with ER-positive/HER2-negative breast cancer and found that peritumoral radiomic information, particularly from a 3-mm region on T2-weighted imaging, contributed to prediction of the 21-gene recurrence score [21]. Collectively, these studies support the concept that the peritumoral region contains quantitative imaging information related to tumor phenotype, immune context, and prognostic characteristics. However, their primary objective has generally been to develop or improve predictive models for predefined clinical or molecular endpoints.

The present study differs from this predictive paradigm. Rather than selecting radiomic features according to their association with a molecular subtype, treatment response, or prognostic outcome, we examined the relative position of the peritumoral microenvironment within an intra-patient radiomic feature space. Specifically, peritumoral-to-normal distances were interpreted against the bilateral normal-appearing parenchymal distance ($d_{IC}$). This design addresses a different question: whether the radiomic deviation surrounding the tumor is greater than the level of variation already present between normal-appearing tissues within the same individual. The consistency of this relationship across four mathematically distinct distance metrics suggests that the observed effect is not dependent on a single similarity formulation.

Studies examining breast tissue outside the tumor provide additional context for this approach. Arefan et al. showed that quantitative background parenchymal enhancement measured in both the ipsilateral and contralateral breasts was associated with recurrence-risk classification and provided complementary information when combined with tumor radiomics [18]. Similarly, Lo Gullo et al. demonstrated that radiomic features extracted exclusively from fibroglandular tissue of the contralateral unaffected breast contained information associated with triple-negative breast cancer phenotype [19]. These findings emphasize that radiologically normal or unaffected breast tissue should not necessarily be considered quantitatively uninformative. Instead, breast parenchyma may contain patient-specific imaging characteristics that are relevant when interpreting tumor-associated or peritumoral radiomic alterations.

This distinction is central to the interpretation of our findings. If normal-appearing breast parenchyma itself exhibits meaningful radiomic heterogeneity, comparing the peritumoral region with a single normal ROI without accounting for background within-patient variability may overstate or underestimate the magnitude of peritumoral alteration. By explicitly quantifying the ipsilateral-to-contralateral distance and using it as an internal reference, the present framework places the peritumoral deviation within the patient’s own bilateral radiomic context. From a radiological perspective, this bilateral context is particularly relevant because background parenchymal enhancement and fibroglandular tissue architecture may vary both between patients and between the two breasts. Accordingly, the use of ipsilateral and contralateral normal-appearing parenchyma provides a patient-specific imaging context within which the magnitude of peritumoral radiomic deviation can be interpreted. Thus, our results complement previous predictive peritumoral radiomics studies by providing a direct tissue-characterization perspective rather than another endpoint-specific classification model.

### 4.3. Significance of the Within-Patient Bilateral Reference Framework

A central methodological feature of the present study is the use of the ipsilateral-to-contralateral normal-appearing parenchymal distance ($d_{IC}$) as an internal patient-specific reference. Rather than assuming that normal-appearing breast tissue represents a fixed or homogeneous radiomic state, this framework explicitly acknowledges that measurable variation may exist between the two breasts even in regions without radiologically apparent abnormality.

This distinction is particularly important in radiomic analyses, in which quantitative feature values may be influenced by patient-specific tissue composition, background parenchymal characteristics, imaging-related variation, and other sources of biological heterogeneity. A peritumoral-to-normal distance considered in isolation does not indicate whether the observed difference is unusually large for that individual. By contrast, comparison with $d_{IC}$ provides an internal scale against which the magnitude of the peritumoral deviation can be interpreted.

In the present cohort, $d_{IC}$ showed the lowest mean distance among the principal tissue-pair comparisons across all four distance metrics. Moreover, both $d_{MI}$ and $d_{MC}$ consistently exceeded $d_{IC}$ at the group level and in the majority of individual patients. These findings show that, in the present cohort, the peritumoral-to-normal distances were greater than the distance between the two selected normal-appearing reference regions. Importantly, this interpretation does not require either normal-appearing region to be considered histologically “healthy” or biologically identical; $d_{IC}$ is used here as a patient-specific reference distance rather than as an estimate of the full distribution of bilateral parenchymal variability.

The intra-patient design also reduces the contribution of between-patient heterogeneity to the primary paired comparisons. Specifically, the bilateral normal-appearing tissue pair within each patient provides a patient-specific internal reference scale against which the magnitude of peritumoral deviation can be evaluated. This differs conceptually from comparisons based solely on pooled normal tissue from unrelated individuals, in which inter-individual differences may contribute substantially to the observed feature-space separation.

From a breast MRI perspective, normal-appearing parenchyma in the two breasts should not necessarily be expected to exhibit identical quantitative imaging characteristics. Bilateral differences may arise from variation in fibroglandular tissue distribution, background parenchymal enhancement, hormonal responsiveness, menopausal status, and other patient-specific parenchymal characteristics. These factors were not measured separately in the present study and therefore cannot be identified as specific sources of the observed $d_{IC}$ values. Nevertheless, their potential contribution further supports the use of the bilateral normal-appearing tissue distance as an empirical patient-specific reference rather than assuming perfect bilateral radiomic symmetry.

The use of both ipsilateral and contralateral normal-appearing parenchyma provides an additional advantage. If the peritumoral region had differed only from the contralateral breast, the observed effect could potentially have reflected laterality, breast-specific tissue composition, or characteristics of the tumor-bearing breast as a whole. However, the consistent elevation of both $d_{MI}$ and $d_{MC}$ relative to $d_{IC}$ indicates that the observed peritumoral-to-normal radiomic distances were not captured by the bilateral normal-appearing parenchymal reference distance alone.

In routine breast MRI interpretation, bilateral comparison with the contralateral breast provides useful anatomical and imaging context for evaluating parenchymal symmetry and identifying abnormalities in either breast. The present framework extends this comparative principle quantitatively by incorporating the contralateral normal-appearing parenchyma into a within-patient radiomic reference. However, this should not be interpreted as implying that the contralateral breast represents a fixed physiological standard or that $d_{IC}$ constitutes a clinically established threshold.

Accordingly, $d_{IC}$ should not be interpreted as a universal physiological threshold or as a histological gold standard for normal breast tissue. It is instead a patient-specific internal reference that quantifies the observed bilateral radiomic difference under the imaging conditions of the present study. Future studies incorporating larger cohorts, repeated imaging, independent tissue characterization, and explicit assessment of factors such as breast density and background parenchymal enhancement will be required to determine how stable this reference is across acquisition protocols, patient populations, and clinical settings.

### 4.4. Robustness Across Distance Metrics, Covariance Estimation, and Feature Composition

An important feature of the present study is the consistency of the principal finding across four mathematically distinct distance formulations. Standardized Euclidean, Manhattan, cosine, and Mahalanobis distances emphasize different aspects of the radiomic feature space. Euclidean distance summarizes overall geometric separation after feature standardization, Manhattan distance reflects cumulative absolute feature-wise differences, cosine distance captures differences in feature-profile orientation within the standardized feature space, and Mahalanobis distance additionally incorporates inter-feature covariance. Despite these differences, both $d_{MI}>d_{IC}$ and $d_{MC}>d_{IC}$ were observed consistently across all four metrics.

This cross-metric agreement reduces the likelihood that the principal finding is dependent on a single distance definition. In the primary 101-feature analysis, all eight paired comparisons remained statistically significant after Benjamini–Hochberg correction, with large matched-pairs rank-biserial effect sizes and preservation of the expected direction in the majority of individual patients. However, these comparisons should not be interpreted as eight independent replications, because the two paired contrasts share the same $d_{IC}$ reference and the four distance metrics are mathematically related. Rather, their concordance provides evidence that the principal relationship is preserved across complementary formulations of radiomic dissimilarity.

The feature-composition sensitivity analyses provided an additional assessment of whether the principal finding could be explained primarily by ROI morphology or by selected texture features that are sensitive to ROI size. After exclusion of all 13 retained shape features, both primary comparisons remained positive and statistically significant across all four distance metrics, although effect sizes were moderately attenuated. This reduction indicates that explicit morphological information contributed substantially to the magnitude of the overall multivariate separation, but did not fully account for the principal radiomic relationship. Because the shape descriptors were derived from single-slice masks and therefore encode in-plane region geometry, which differs between the four ROI types by construction, the shape-free 88-feature analysis provides the more appropriate basis for interpreting the principal finding. Accordingly, greater interpretive emphasis is placed on the 88-feature shape-free analysis, because it removes explicit descriptors of ROI geometry; however, this analysis does not eliminate potential dependence on ROI size or texture-sampling stability.

The normalized-feature replacement analysis yielded a similar conclusion. Replacing five non-normalized non-uniformity measures with their normalized PyRadiomics counterparts within the shape-free feature space preserved the direction and statistical significance of all eight paired comparisons. The Mahalanobis effect sizes were particularly stable under this modification. Taken together, these findings indicate that the observed peritumoral deviation was not solely attributable to explicit shape information or to the evaluated non-normalized non-uniformity features. Thus, although exclusion of the normalized variants could in principle have introduced systematic weighting toward ROI-size-sensitive absolute measures, the replacement analysis did not produce a reversal or loss of statistical significance in any of the eight primary paired comparisons. Nevertheless, these analyses should be regarded as sensitivity assessments rather than as evidence that all possible effects of ROI geometry or size have been eliminated.

A further consideration concerns ROI size rather than ROI shape. This asymmetry is directly relevant to the distance comparisons: $d_{IC}$ is calculated between two 10-mm circular reference ROIs with matched nominal size and similar sampling geometry, whereas $d_{MI}$ and $d_{MC}$ compare the larger, irregular peritumoral region with a smaller fixed-size reference ROI. Consequently, part of the observed excess of $d_{MI}$ and $d_{MC}$ over $d_{IC}$ may reflect differences in ROI size, shape, and texture-sampling stability rather than tissue composition alone. The reference parenchymal regions were smaller than the tumor and peritumoral regions, and several retained first-order and texture descriptors, including energy-type measures and absolute non-uniformity measures, scale with the number of analyzed voxels. In addition, texture matrices estimated from smaller regions are subject to greater sampling variability, which may itself contribute to measured distances. The feature-composition analyses reported here reduce, but do not eliminate, these influences, because they were not accompanied by an explicit size-matched resampling procedure. A formal size-matched analysis, in which peritumoral and tumor regions are subsampled to the voxel count of the reference ROIs, would be required to separate size-related from tissue-related contributions, and we regard this as a necessary component of future work.

The Mahalanobis analysis required particular attention because the dimensionality of the radiomic feature space exceeded the number of contralateral reference observations. The empirical covariance matrix was rank-deficient (55/101) and extremely ill-conditioned, with a condition number of approximately $4.61\times 10^{18}$. Ledoit–Wolf shrinkage substantially improved numerical stability, reducing the condition number to approximately 173.65 while preserving the direction and magnitude of the principal effects. Because the Mahalanobis metric was defined using covariance estimated from the contralateral reference compartment, its absolute distances are conditional not only on the covariance estimator but also on the selected reference population. The present covariance-sensitivity analyses assess robustness to alternative covariance estimators, but they do not establish invariance to using a different tissue compartment or a pooled bilateral normal-appearing reference for covariance estimation.

The stability of the Mahalanobis results across covariance estimators further supports the robustness of the group-level findings. The principal effects remained positive and statistically significant when the Moore–Penrose pseudo-inverse was used and across fixed-shrinkage values ranging from δ=0.05 to δ=1.00. Although the absolute magnitude of Mahalanobis distances changed across covariance formulations, the inferential conclusions remained stable. This distinction is important because absolute Mahalanobis values are inherently dependent on the covariance model.

At the individual-patient level, however, agreement between Mahalanobis formulations was more limited for some distance measures. Spearman correlations between the Moore–Penrose and Ledoit–Wolf estimates were moderate for $d_{MI}$, $d_{MC}$, and $d_{IC}$, indicating that patient-level ordering can depend on the covariance estimator even when the overall group-level direction and inferential conclusions remain stable. Accordingly, the present Mahalanobis-based measures should not be interpreted as validated individual-patient biomarkers on the basis of the current analysis.

The reduced-dimensional PCA analyses provided a further sensitivity assessment. The primary analyses were intentionally performed in the full 101-dimensional standardized feature space to preserve the complete radiomic representation. Nevertheless, when distances were recalculated in lower-dimensional PCA spaces, the same overall direction of effect was broadly maintained. The preservation of the pattern in PCA-3 and PCA-4 spaces suggests that the principal radiomic relationship is also represented within the dominant directions of variance captured by the reduced-dimensional feature space. At the same time, the partial overlap observed among the peritumoral and normal-appearing parenchymal regions in the two-dimensional PCA projection emphasizes that the study does not depend on visually separable clusters in low-dimensional space.

From a radiological perspective, the convergence of the findings across complementary distance formulations, covariance-estimation strategies, reduced-dimensional representations, and alternative feature compositions suggests that the observed peritumoral deviation reflects a broader multivariate radiomic pattern rather than a relationship emerging solely from one particular analytical choice. However, this analytical convergence does not identify the biological mechanism underlying the observed pattern, nor does it eliminate all potential influences of ROI definition, image acquisition, or preprocessing.

### 4.5. Potential Clinical Implications

Although the present study was designed as a radiomic characterization analysis rather than as a diagnostic or predictive modeling study, the proposed within-patient framework may provide a quantitative basis for future clinically oriented investigations. By comparing the peritumoral microenvironment with each patient’s own bilateral normal-appearing parenchymal background, the approach provides an individualized context for interpreting the magnitude of peritumoral radiomic deviation.

Future studies may investigate whether the magnitude of this deviation is associated with clinically relevant tumor characteristics, including molecular subtype, histologic grade, tumor size, lymph-node involvement, treatment response, recurrence, or survival. If validated in larger independent cohorts, peritumoral radiomic deviation could be evaluated as a candidate quantitative imaging measure in studies investigating tumor aggressiveness, local tissue extension, treatment response, recurrence risk, and other clinically relevant outcomes. Importantly, the present study did not assess associations with molecular subtype, histologic grade, lymph-node status, treatment response, recurrence, or survival. Any potential diagnostic, prognostic, predictive, or clinical utility of the proposed radiomic framework therefore remains hypothetical and should be evaluated in appropriately designed and independently validated future studies.

Several steps would be required before clinical translation could be considered, including external validation in larger multi-institutional cohorts, assessment across different MRI scanners and acquisition protocols, evaluation of segmentation reproducibility, and prospective validation. Direct spatial correlation with histopathological findings would also be important for clarifying the biological basis of the observed radiomic deviations. The present results should therefore be regarded as support for a quantitative tissue-characterization framework rather than as evidence for immediate clinical decision making.

### 4.6. Limitations and Future Directions

Several limitations should be considered when interpreting the present findings. First, this retrospective, single-center study included a relatively small cohort of 56 patients, which limits statistical power, particularly for detecting smaller effects or evaluating subgroup-specific patterns, and restricts the external generalizability of the findings. The requirement for complete bilateral breast MRI and suitable normal-appearing reference regions may further limit generalizability. Although the within-patient paired design reduces inter-individual variability, it does not overcome the limitations associated with the relatively small sample size and single-center sampling. Larger, independent, multi-institutional cohorts will therefore be required to confirm the reproducibility of the proposed within-patient framework and to evaluate potential differences across clinical, histopathological, and molecular subgroups.

Second, radiomic feature extraction was based on single axial slice ROI masks rather than full volumetric representations. Although the initial tumor delineation was performed volumetrically, the masks supplied to the final radiomic extraction pipeline occupied a single axial slice. Consequently, the present analysis does not capture the complete three-dimensional spatial heterogeneity of the tumor, peritumoral microenvironment, or reference parenchyma and may be influenced by the selected slice. Future studies should compare this approach with fully volumetric three-dimensional radiomic analysis.

Third, the geometry and sampling strategy of the four ROI types were not identical, and this is the most important interpretive constraint of the present analysis. The ipsilateral and contralateral reference regions were defined as circular ROIs of fixed 10 mm diameter, whereas the tumor and peritumoral regions followed lesion-related boundaries and were therefore substantially larger and irregular in outline. Two consequences follow. First, the shape descriptors retained in the primary feature set differ between region types by construction, and in the present single-slice setting they describe in-plane cross-sectional geometry rather than volumetric morphology. Second, several intensity and texture descriptors depend on the number of analyzed voxels, either through direct scaling or through the reduced stability of texture matrices estimated from smaller regions. The shape-free, feature-family, and normalized-feature analyses reported in Appendix D show that the direction of the effect is preserved under several alternative feature compositions, but they are not equivalent to a size-matched design. Accordingly, the magnitude of the reported distances should not be interpreted as a geometry-independent measure of tissue alteration, and future work should incorporate volumetric segmentation together with explicit size-matched or voxel-count-controlled comparisons.

Fourth, the ipsilateral and contralateral reference regions were defined as normal-appearing breast parenchyma on MRI rather than histologically confirmed normal tissue. Microscopic disease or other subvisual tissue alterations therefore cannot be excluded. Accordingly, $d_{IC}$ should be interpreted as an imaging-derived within-patient bilateral reference distance rather than as a physiological or histopathological standard or as a complete measure of bilateral parenchymal variability.

Fifth, although all examinations were acquired using the same 1.5-T MRI system and an institutional clinical breast MRI protocol, radiomic measurements may remain sensitive to acquisition, reconstruction, spatial sampling, and signal-intensity characteristics. Such dependencies are well recognized as important sources of variability and reproducibility concerns in radiomics, including in MRI-based feature extraction [29,30]. Features were extracted using the native image geometry without spatial resampling or image-level intensity normalization. Native in-plane voxel spacing ranged from 0.566 to 0.879 mm and slice thickness from 0.8 to 1.8 mm. Feature-wise standardization does not correct these image-level differences. Consequently, the absolute magnitudes of $d_{MI}$, $d_{MC}$, and $d_{IC}$ may partly reflect acquisition- and preprocessing-related variability in addition to differences among the evaluated tissue regions. Although the within-patient design reduces this source of confounding because all ROIs contributing to a patient’s distance measures originate from the same examination and post-contrast phase, it does not ensure that distance magnitudes are independent of voxel geometry or signal-intensity scaling across patients. The stability of the proposed relationships under spatial resampling, intensity harmonization, and multi-scanner or multi-protocol conditions therefore remains to be established.

Sixth, a single predefined 5-mm peritumoral expansion was evaluated. The present study therefore does not establish the optimal spatial extent for characterizing peritumoral radiomic deviation. In addition, partial-volume or contrast-related edge effects immediately adjacent to the tumor boundary were not specifically assessed. Future studies should evaluate multiple predefined peritumoral margins and, where appropriate, exclusion zones adjacent to the tumor boundary.

Seventh, manual ROI delineation may introduce reader-related variability. Reproducibility was assessed in a randomly selected subset of 20 patients using interobserver and intraobserver ICC analyses, and all 101 retained radiomic features met the prespecified ICC threshold of 0.75. Nevertheless, this assessment involved a limited subset and readers from a single institution. Reproducibility across institutions, readers with different experience levels, and alternative segmentation strategies remains to be determined.

Eighth, the Mahalanobis analysis was conducted in a high-dimensional setting in which the number of radiomic features exceeded the number of contralateral reference observations. Ledoit–Wolf shrinkage was therefore used for the primary covariance estimate. Although the group-level findings were preserved across alternative covariance formulations, patient-level ordering showed only moderate agreement for some distance measures. Accordingly, the Mahalanobis-derived distances should not be interpreted as validated individual-patient biomarkers.

Finally, the study was designed to characterize radiomic relationships among the evaluated regions rather than to evaluate diagnostic, prognostic, or treatment-response endpoints. Associations with molecular subtype, histologic grade, lymph-node status, treatment response, recurrence, and survival were not evaluated. The observed peritumoral deviation should therefore not be interpreted as evidence of tumor aggressiveness, microscopic extension, treatment sensitivity, recurrence risk, survival outcome, or a discrete biological tissue class. Future prospective studies should determine whether patient-specific deviation measures are associated with clinically relevant outcomes and whether they provide information beyond conventional imaging features and tumor-centered radiomic models.

## 5. Conclusions

In this retrospective single-center cohort, both the peritumoral-to-ipsilateral ($d_{MI}$) and peritumoral-to-contralateral ($d_{MC}$) radiomic distances exceeded the ipsilateral-to-contralateral normal-appearing parenchymal reference distance ($d_{IC}$) across standardized Euclidean, Manhattan, cosine, and Ledoit–Wolf Mahalanobis formulations. The principal relationship remained statistically significant after multiple-testing correction and was broadly preserved across the evaluated covariance, dimensionality-reduction, and feature-composition sensitivity analyses. Although removal of explicit shape features attenuated effect magnitude, the direction and statistical significance of the primary comparisons were maintained.

The use of $d_{IC}$ as an internal patient-specific reference provides a quantitative framework for contextualizing peritumoral radiomic deviation relative to each patient’s own bilateral normal-appearing parenchymal reference distance. However, the present findings are based on single-slice radiomic analysis of regions that differ in size and geometry by design, and should not be interpreted as evidence of a specific biological mechanism, microscopic tumor extension, prognostic value, or clinical utility. In particular, the contribution of ROI size and sampling geometry was reduced but not formally eliminated by the reported sensitivity analyses. Validation in larger multi-institutional cohorts using full-volume three-dimensional radiomics, standardized imaging and preprocessing strategies, and correlation with histopathological and clinical outcomes will be required to establish the reproducibility and biological relevance of this within-patient reference framework.

## Figures and Tables

**Table 1:** Descriptive statistics of patient-specific radiomic distances in the final 101-feature analysis. Values are presented as mean ± standard deviation.

Distance Metric	$d_{\mathrm{MT}}$	$d_{\mathrm{MI}}$	$d_{\mathrm{MC}}$	$d_{\mathrm{IC}}$	MSI
Standardized Euclidean	15.84±6.65	9.06±3.25	9.67±3.74	5.76±2.90	−6.78±5.59
Manhattan	131.67±57.52	67.17±25.46	70.60±30.39	40.78±22.35	−64.51±50.51
Cosine	1.139±0.390	0.742±0.417	0.775±0.367	0.357±0.300	−0.397±0.625
Mahalanobis (Ledoit–Wolf)	17.52±9.98	9.29±2.84	9.47±2.63	4.91±2.00	−8.23±9.69

**Table 2:** Primary paired comparisons of peritumoral-to-normal radiomic distances against the within-patient bilateral normal-appearing parenchymal reference distance. Median paired differences are reported with the 25th and 75th percentiles (Q1–Q3).

Metric	Comparison	Mean Diff.	Median Diff. [Q1–Q3]	*W*	BH-Adjusted *q*	$r_{\mathrm{rb}}$ (95% CI)
Euclidean	$d_{MI}-d_{IC}$	3.29	3.75 [1.30, 5.66]	179	$5.07\times 10^{-7}$	0.776 (0.581–0.926)
	$d_{MC}-d_{IC}$	3.91	3.56 [1.51, 5.87]	78	$1.14\times 10^{-8}$	0.902 (0.777–0.984)
Manhattan	$d_{MI}-d_{IC}$	26.39	31.00 [11.71, 43.06]	178	$5.07\times 10^{-7}$	0.777 (0.580–0.924)
	$d_{MC}-d_{IC}$	29.82	26.20 [10.99, 46.86]	117	$4.44\times 10^{-8}$	0.853 (0.702–0.961)
Cosine	$d_{MI}-d_{IC}$	0.384	0.394 [0.092, 0.693]	200	$1.07\times 10^{-6}$	0.749 (0.541–0.909)
	$d_{MC}-d_{IC}$	0.417	0.335 [0.158, 0.706]	107	$3.47\times 10^{-8}$	0.866 (0.718–0.972)
Mahalanobis	$d_{MI}-d_{IC}$	4.38	4.30 [3.06, 6.21]	44	$3.09\times 10^{-9}$	0.945 (0.851–1.000)
	$d_{MC}-d_{IC}$	4.56	4.45 [2.81, 5.90]	9	$9.82\times 10^{-10}$	0.989 (0.957–1.000)

## Data Availability

The imaging data supporting the findings of this study are not publicly available because of institutional restrictions and patient privacy requirements. The anonymized, de-identified radiomic feature matrix and the analysis code used to generate the reported results are available from the corresponding author upon reasonable request.

## Abbreviations

BPE: background parenchymal enhancement; BH: Benjamini–Hochberg; DCE-MRI: dynamic contrast-enhanced magnetic resonance imaging; DWI: diffusion-weighted imaging; GLCM: gray-level co-occurrence matrix; GLDM: gray-level dependence matrix; GLRLM: gray-level run-length matrix; GLSZM: gray-level size-zone matrix; IBSI: Image Biomarker Standardisation Initiative; ICC: intraclass correlation coefficient; MSI: microenvironment similarity index; NGTDM: neighboring gray-tone difference matrix; PCA: principal component analysis; pCR: pathological complete response; ROI: region of interest; TME: tumor microenvironment.

## Appendix A. Parametric Sensitivity Analysis

As a complementary analysis, paired *t*-tests were performed for the eight primary comparisons. The direction of the effects was consistent with the primary Wilcoxon signed-rank analyses across all distance metrics. All 95% confidence intervals for the mean paired differences remained above zero.

**Table A1:** Parametric sensitivity analysis for the primary paired radiomic distance comparisons.

Metric	Comparison	Mean Diff.	95% CI	*p*-Value	Cohen’s $d_{z}$
Euclidean	$d_{MI}-d_{IC}$	3.29	2.33–4.26	$6.79\times 10^{-9}$	0.915
	$d_{MC}-d_{IC}$	3.91	2.96–4.85	$3.27\times 10^{-11}$	1.104
Manhattan	$d_{MI}-d_{IC}$	26.39	18.59–34.19	$8.57\times 10^{-9}$	0.906
	$d_{MC}-d_{IC}$	29.82	21.62–38.03	$1.30\times 10^{-9}$	0.973
Cosine	$d_{MI}-d_{IC}$	0.384	0.258–0.510	$1.03\times 10^{-7}$	0.818
	$d_{MC}-d_{IC}$	0.417	0.308–0.526	$3.15\times 10^{-10}$	1.023
Mahalanobis	$d_{MI}-d_{IC}$	4.38	3.56–5.20	$3.95\times 10^{-15}$	1.436
	$d_{MC}-d_{IC}$	4.56	3.84–5.28	$5.53\times 10^{-18}$	1.697

## Appendix B. Mahalanobis Covariance Sensitivity Analysis

To evaluate whether the Mahalanobis-based findings depended on the covariance estimation strategy, the primary Ledoit–Wolf formulation was compared with an empirical covariance matrix using the Moore–Penrose pseudo-inverse and with a series of fixed-shrinkage covariance estimators. Across all covariance formulations, the mean paired differences remained positive for both $d_{MI}-d_{IC}$ and $d_{MC}-d_{IC}$ (Table A2).

The direction of the primary findings was preserved over the complete fixed-shrinkage range (δ=0.05–1.00). For $d_{MI}-d_{IC}$, rank-biserial correlations ranged from 0.776 to 0.960 across the fixed-shrinkage settings, whereas those for $d_{MC}-d_{IC}$ ranged from 0.902 to 0.991. The Moore–Penrose formulation similarly produced strong positive effects. These findings indicate that the observed peritumoral deviation beyond bilateral normal-appearing parenchymal reference distance was not dependent on a specific covariance regularization strength.

**Table A2:** Mahalanobis covariance-estimation sensitivity analysis. Reported *p*-values correspond to two-sided Wilcoxon signed-rank tests.

Estimator	Comparison	Mean Diff.	*W*	*p*-Value	$r_{\mathrm{rb}}$	Positive/56
Ledoit–Wolf (δ=0.2082)	$d_{MI}-d_{IC}$	4.38	44	$7.73\times 10^{-10}$	0.945	53
	$d_{MC}-d_{IC}$	4.56	9	$1.23\times 10^{-10}$	0.989	55
Moore–Penrose	$d_{MI}-d_{IC}$	42.56	3	$8.88\times 10^{-11}$	0.996	55
	$d_{MC}-d_{IC}$	42.28	5	$9.89\times 10^{-11}$	0.994	55
Fixed δ=0.05	$d_{MI}-d_{IC}$	7.33	32	$4.15\times 10^{-10}$	0.960	53
	$d_{MC}-d_{IC}$	7.39	7	$1.10\times 10^{-10}$	0.991	55
Fixed δ=0.10	$d_{MI}-d_{IC}$	5.64	38	$5.67\times 10^{-10}$	0.952	52
	$d_{MC}-d_{IC}$	5.77	8	$1.16\times 10^{-10}$	0.990	55
Fixed δ=0.25	$d_{MI}-d_{IC}$	4.08	47	$9.01\times 10^{-10}$	0.941	53
	$d_{MC}-d_{IC}$	4.27	10	$1.29\times 10^{-10}$	0.987	54
Fixed δ=0.50	$d_{MI}-d_{IC}$	3.31	72	$3.18\times 10^{-9}$	0.910	51
	$d_{MC}-d_{IC}$	3.58	8	$1.16\times 10^{-10}$	0.990	54
Fixed δ=0.75	$d_{MI}-d_{IC}$	3.10	101	$1.30\times 10^{-8}$	0.873	50
	$d_{MC}-d_{IC}$	3.48	14	$1.60\times 10^{-10}$	0.982	53
Fixed δ=1.00	$d_{MI}-d_{IC}$	4.11	179	$4.44\times 10^{-7}$	0.776	47
	$d_{MC}-d_{IC}$	4.88	78	$4.28\times 10^{-9}$	0.902	50

## Appendix C. Principal Component Analysis

Principal component analysis (PCA) was performed on the standardized 101-feature radiomic matrix as a complementary reduced-dimensional analysis. PCA was not used for the primary statistical inference; rather, it was employed to examine whether the principal distance relationships were preserved when the radiomic feature space was represented by a smaller number of orthogonal components. Separate PCA representations retaining the first two, three, and four principal components were evaluated. For each reduced-dimensional representation, standardized Euclidean, Manhattan, cosine, and Mahalanobis distances were recalculated using the corresponding PCA scores. For the Mahalanobis distance, the empirical covariance matrix was estimated exclusively from the 56 contralateral normal-appearing parenchymal observations in the respective PCA space. Because the resulting 2×2, 3×3, and 4×4 covariance matrices were full rank and directly invertible, the conventional matrix inverse was used without shrinkage regularization or pseudo-inversion.

The first principal component accounted for 50.76% of the total variance. The first two, three, and four components cumulatively explained 62.95%, 74.42%, and 80.49% of the total variance, respectively (Table A3).

**Table A3:** Explained variance of the first four principal components.

Component	Explained Variance (%)	Cumulative Variance (%)
PC1	50.76	50.76
PC2	12.18	62.95
PC3	11.48	74.42
PC4	6.07	80.49

Patient-specific radiomic distances were recalculated in the two-, three-, and four-component PCA spaces. Positive mean differences between the peritumoral-to-normal distances and the bilateral normal-appearing parenchymal reference were retained across all evaluated PCA dimensions and distance metrics (Table A4).

In the two-component representation, seven of the eight exploratory paired comparisons reached a nominal two-sided Wilcoxon significance level of p<0.05. The only exception was the cosine-distance comparison between $d_{MC}$ and $d_{IC}$ (p=0.078), although the mean paired difference remained positive. With three principal components, all eight comparisons were statistically significant, with Wilcoxon *p*-values ranging from $3.43\times 10^{-7}$ to $4.38\times 10^{-5}$. The same pattern was strengthened in the four-component representation, in which all eight comparisons remained significant and positive. These reduced-dimensional findings were therefore broadly consistent with the direction of the primary full-feature-space analysis, particularly when at least three principal components were retained.

The two-dimensional PCA projection is shown in Figure A1. Tumor observations demonstrated a visually distinct tendency along the first principal component, whereas the peritumoral microenvironment and the two normal-appearing parenchymal regions showed substantial overlap. Therefore, the PCA visualization was interpreted as an exploratory representation of the global radiomic structure rather than as evidence of class separation. Importantly, the primary analyses were conducted in the full 101-dimensional standardized feature space and did not rely on visual separation in the reduced-dimensional PCA representation.

**Table A4:** Exploratory paired comparisons in reduced-dimensional PCA spaces. *p*-values correspond to two-sided Wilcoxon signed-rank tests and are presented as complementary, unadjusted sensitivity results.

PCA Space	Metric	$\Delta_{\mathrm{MI}}$	$W_{\mathrm{MI}}$	$p_{\mathrm{MI}}$	$\Delta_{\mathrm{MC}}$	$W_{\mathrm{MC}}$	$p_{\mathrm{MC}}$
PCA-2	Euclidean	1.541	344	$2.13\times 10^{-4}$	1.399	457	$5.41\times 10^{-3}$
	Manhattan	1.917	364	$4.00\times 10^{-4}$	1.731	466	$6.77\times 10^{-3}$
	Cosine	0.188	526	$2.65\times 10^{-2}$	0.128	582	$7.81\times 10^{-2}$
	Mahalanobis	0.316	444	$3.88\times 10^{-3}$	0.353	456	$5.28\times 10^{-3}$
PCA-3	Euclidean	2.702	246	$6.71\times 10^{-6}$	3.067	195	$8.71\times 10^{-7}$
	Manhattan	4.282	235	$4.38\times 10^{-6}$	4.561	211	$1.68\times 10^{-6}$
	Cosine	0.412	297	$4.38\times 10^{-5}$	0.424	227	$3.20\times 10^{-6}$
	Mahalanobis	0.817	269	$1.60\times 10^{-5}$	0.985	173	$3.43\times 10^{-7}$
PCA-4	Euclidean	2.897	233	$4.05\times 10^{-6}$	3.413	164	$2.32\times 10^{-7}$
	Manhattan	4.907	245	$6.46\times 10^{-6}$	5.773	184	$5.49\times 10^{-7}$
	Cosine	0.413	256	$9.82\times 10^{-6}$	0.450	166	$2.53\times 10^{-7}$
	Mahalanobis	0.883	255	$9.45\times 10^{-6}$	1.105	143	$9.15\times 10^{-8}$

## Appendix D. Feature-Composition Sensitivity Analyses

Additional sensitivity analyses were performed to assess the robustness of the principal findings to radiomic feature composition. These analyses included: (i) exclusion of all shape features, (ii) separate analysis of individual radiomic feature families, and (iii) replacement of selected non-normalized non-uniformity features with their normalized PyRadiomics counterparts. The analytical and statistical procedures were identical to those used in the primary analysis, with feature-wise standardization refitted independently within each evaluated feature space.

### Appendix D.1. Shape-Free Sensitivity Analysis

After exclusion of all 13 shape features retained in the primary dataset, 88 first-order and texture features remained. Both primary paired differences remained positive across all four distance metrics, and all eight comparisons remained statistically significant after Benjamini–Hochberg correction (Table A5). Although effect sizes were moderately reduced relative to the primary 101-feature analysis, the direction and patient-level consistency of the findings were preserved.

**Table A5:** Shape-free sensitivity analysis using 88 first-order and texture features. *q*-values correspond to Benjamini–Hochberg-adjusted two-sided Wilcoxon signed-rank tests. Rank-biserial correlations are reported with 95% bootstrap confidence intervals.

Metric	Comparison	Mean Diff.	*W*	*q*-Value	$r_{\mathrm{rb}}$ (95% CI)	Positive/56	Cohen’s $d_{z}$
Euclidean	$d_{MI}-d_{IC}$	2.879	219	$3.10\times 10^{-6}$	0.726 (0.511–0.898)	44/56	0.810
Euclidean	$d_{MC}-d_{IC}$	3.415	119	$9.25\times 10^{-8}$	0.851 (0.692–0.966)	50/56	0.974
Manhattan	$d_{MI}-d_{IC}$	20.980	239	$5.85\times 10^{-6}$	0.701 (0.470–0.885)	44/56	0.773
Manhattan	$d_{MC}-d_{IC}$	23.388	184	$8.78\times 10^{-7}$	0.769 (0.569–0.921)	46/56	0.802
Cosine	$d_{MI}-d_{IC}$	0.367	252	$8.44\times 10^{-6}$	0.684 (0.454–0.872)	44/56	0.739
Cosine	$d_{MC}-d_{IC}$	0.393	128	$9.25\times 10^{-8}$	0.840 (0.665–0.972)	50/56	0.905
Mahalanobis	$d_{MI}-d_{IC}$	2.952	127	$9.25\times 10^{-8}$	0.841 (0.673–0.965)	49/56	1.090
Mahalanobis	$d_{MC}-d_{IC}$	3.188	53	$9.79\times 10^{-9}$	0.934 (0.841–0.996)	52/56	1.294

### Appendix D.2. Feature-Family Sensitivity Analysis

To determine whether the principal effect was concentrated within a specific class of radiomic features, each feature family was analyzed independently. Across shape, first-order, GLCM, GLRLM, GLSZM, GLDM, and NGTDM feature spaces, both primary paired differences remained positive for every distance metric. All family-specific comparisons remained statistically significant after the corresponding within-feature-space Benjamini–Hochberg correction (Table A6).

Across feature families, shape features generally produced the largest effects, whereas first-order features yielded the smallest effect sizes. Importantly, however, no feature family showed reversal of the principal direction of effect. Even in the weakest family-specific comparison, the paired difference remained positive and statistically significant after correction for multiple testing.

**Table A6:** Feature-family sensitivity analyses. $\Delta_{MI}=d_{MI}-d_{IC}$ and $\Delta_{MC}=d_{MC}-d_{IC}$. *q*-values correspond to Benjamini–Hochberg-adjusted two-sided Wilcoxon signed-rank tests performed within each feature-family analysis.

Family	Metric	$\Delta_{\mathrm{MI}}$	$q_{\mathrm{MI}}$	$r_{\mathrm{MI}}$	Pos.	$d_{z,\mathrm{MI}}$	$\Delta_{\mathrm{MC}}$	$q_{\mathrm{MC}}$	$r_{\mathrm{MC}}$	Pos.	$d_{z,\mathrm{MC}}$
Shape	Euclidean	1.688	$9.13\times 10^{-8}$	0.830	46/56	1.008	1.939	$2.58\times 10^{-9}$	0.932	50/56	1.284
Shape	Manhattan	5.409	$9.13\times 10^{-8}$	0.825	47/56	0.985	6.435	$1.96\times 10^{-9}$	0.946	51/56	1.343
Shape	Cosine	0.518	$6.78\times 10^{-7}$	0.763	48/56	0.778	0.558	$9.13\times 10^{-8}$	0.828	50/56	0.949
Shape	Mahalanobis	5.033	$4.91\times 10^{-10}$	0.989	54/56	1.499	5.144	$4.91\times 10^{-10}$	0.999	55/56	1.609
First-order	Euclidean	0.965	$2.21\times 10^{-4}$	0.573	44/56	0.532	1.466	$1.94\times 10^{-6}$	0.793	47/56	0.837
First-order	Manhattan	3.593	$1.74\times 10^{-4}$	0.588	42/56	0.578	4.831	$2.02\times 10^{-6}$	0.772	45/56	0.809
First-order	Cosine	0.322	$1.06\times 10^{-4}$	0.613	42/56	0.596	0.383	$7.49\times 10^{-6}$	0.711	43/56	0.727
First-order	Mahalanobis	0.753	$1.10\times 10^{-3}$	0.501	40/56	0.421	1.138	$5.07\times 10^{-6}$	0.732	44/56	0.674
GLCM	Euclidean	1.285	$2.51\times 10^{-4}$	0.569	44/56	0.577	1.349	$1.25\times 10^{-4}$	0.625	41/56	0.611
GLCM	Manhattan	4.972	$2.23\times 10^{-4}$	0.585	41/56	0.566	4.664	$5.94\times 10^{-4}$	0.528	37/56	0.502
GLCM	Cosine	0.308	$2.23\times 10^{-4}$	0.585	43/56	0.572	0.283	$2.51\times 10^{-4}$	0.568	44/56	0.524
GLCM	Mahalanobis	1.799	$8.00\times 10^{-8}$	0.862	48/56	1.012	1.969	$2.19\times 10^{-8}$	0.914	50/56	1.159
GLRLM	Euclidean	1.305	$2.09\times 10^{-6}$	0.734	46/56	0.786	1.473	$5.47\times 10^{-7}$	0.810	44/56	0.892
GLRLM	Manhattan	3.886	$2.09\times 10^{-6}$	0.733	46/56	0.782	4.159	$1.18\times 10^{-6}$	0.761	45/56	0.780
GLRLM	Cosine	0.421	$7.82\times 10^{-6}$	0.687	43/56	0.722	0.430	$7.05\times 10^{-7}$	0.791	45/56	0.854
GLRLM	Mahalanobis	1.109	$1.01\times 10^{-6}$	0.772	46/56	0.871	1.208	$2.33\times 10^{-7}$	0.852	47/56	1.004
GLSZM	Euclidean	1.367	$2.77\times 10^{-6}$	0.724	46/56	0.776	1.605	$8.79\times 10^{-8}$	0.860	48/56	0.942
GLSZM	Manhattan	3.770	$2.77\times 10^{-6}$	0.728	46/56	0.772	4.356	$3.13\times 10^{-7}$	0.806	46/56	0.831
GLSZM	Cosine	0.403	$7.24\times 10^{-6}$	0.689	45/56	0.719	0.434	$1.23\times 10^{-7}$	0.840	48/56	0.916
GLSZM	Mahalanobis	1.281	$1.79\times 10^{-6}$	0.748	46/56	0.789	1.476	$8.62\times 10^{-8}$	0.878	46/56	0.917
GLDM	Euclidean	1.387	$9.96\times 10^{-7}$	0.756	45/56	0.819	1.597	$5.22\times 10^{-8}$	0.873	48/56	0.984
GLDM	Manhattan	3.900	$9.96\times 10^{-7}$	0.759	45/56	0.820	4.322	$1.67\times 10^{-7}$	0.823	45/56	0.881
GLDM	Cosine	0.444	$2.84\times 10^{-6}$	0.719	43/56	0.805	0.454	$5.33\times 10^{-8}$	0.862	50/56	0.967
GLDM	Mahalanobis	1.274	$7.73\times 10^{-7}$	0.773	45/56	0.864	1.397	$4.82\times 10^{-8}$	0.893	49/56	1.046
NGTDM	Euclidean	0.549	$3.04\times 10^{-5}$	0.645	39/56	0.631	0.641	$1.54\times 10^{-6}$	0.759	44/56	0.646
NGTDM	Manhattan	0.860	$4.69\times 10^{-5}$	0.625	40/56	0.607	1.056	$1.13\times 10^{-6}$	0.777	47/56	0.710
NGTDM	Cosine	0.332	$2.29\times 10^{-5}$	0.660	44/56	0.595	0.418	$4.79\times 10^{-7}$	0.813	47/56	0.764
NGTDM	Mahalanobis	1.256	$2.20\times 10^{-5}$	0.668	42/56	0.701	1.499	$4.79\times 10^{-7}$	0.816	49/56	0.814

### Appendix D.3. Normalized Non-Uniformity Replacement Analysis

To further examine potential sensitivity to ROI-size-dependent texture features, five non-normalized non-uniformity measures were replaced with their normalized PyRadiomics counterparts within the 88-feature shape-free feature space. Both primary comparisons remained positive and statistically significant across all four distance metrics (Table A7).

**Table A7:** Shape-free sensitivity analysis after replacement of five non-normalized non-uniformity features with their normalized PyRadiomics counterparts. *q*-values correspond to Benjamini–Hochberg-adjusted two-sided Wilcoxon signed-rank tests. Rank-biserial correlations are reported with 95% bootstrap confidence intervals.

Metric	Comparison	Mean Diff.	*W*	*q*-Value	$r_{\mathrm{rb}}$ (95% CI)	Positive/56	Cohen’s $d_{z}$
Euclidean	$d_{MI}-d_{IC}$	2.760	246	$8.95\times 10^{-6}$	0.692 (0.470–0.876)	44/56	0.743
Euclidean	$d_{MC}-d_{IC}$	3.119	159	$4.44\times 10^{-7}$	0.801 (0.618–0.937)	47/56	0.829
Manhattan	$d_{MI}-d_{IC}$	20.312	256	$1.12\times 10^{-5}$	0.679 (0.444–0.872)	43/56	0.709
Manhattan	$d_{MC}-d_{IC}$	21.461	244	$8.95\times 10^{-6}$	0.694 (0.467–0.875)	45/56	0.684
Cosine	$d_{MI}-d_{IC}$	0.347	268	$1.54\times 10^{-5}$	0.664 (0.427–0.858)	43/56	0.678
Cosine	$d_{MC}-d_{IC}$	0.352	163	$4.44\times 10^{-7}$	0.796 (0.603–0.946)	49/56	0.778
Mahalanobis	$d_{MI}-d_{IC}$	2.940	128	$1.85\times 10^{-7}$	0.840 (0.670–0.965)	49/56	1.090
Mahalanobis	$d_{MC}-d_{IC}$	3.171	58	$1.26\times 10^{-8}$	0.927 (0.830–0.995)	52/56	1.255

Overall, the persistence of the principal findings after removal of explicit shape information, across independently analyzed radiomic feature families, and after replacement of selected non-normalized non-uniformity features indicates that the observed peritumoral radiomic deviation was not solely dependent on a single category of radiomic features or on the evaluated size-sensitive texture measures.
